# Biochar: a robust and renewable basic carbocatalyst for sustainable synthesis of diversified spirooxindoles

**DOI:** 10.1039/d5ra05293a

**Published:** 2025-10-03

**Authors:** Ali Khoy, Dariush Khalili, Hamid Reza Boostani

**Affiliations:** a Department of Chemistry, College of Sciences, Shiraz University Shiraz 71467-13565 Iran khalili@shirazu.ac.ir; b Department of Soil and Water Engineering, College of Agriculture and Natural Resources of Darab, Shiraz University Darab Iran

## Abstract

In this study, biochars have been utilized as reusable basic carbocatalysts to get quick access to multi-functionalized spirooxindoles using a sustainable approach. Various organic wastes and manure sources were pyrolyzed into corresponding biochars through a pyrolysis-carbonization process. Compelling evidence supporting the basic nature of the synthesized biochars was provided through the potentiometric titration analysis and chemical characteristics. These analyses substantiated the pronounced alkalinity of the biochar derived from cow manure pyrolyzed at 600 °C (CB600), positioning it as the optimal carbocatalyst in this investigation. The catalytic performance of the biochars was subsequently assessed in the three-component one-pot synthesis of spirooxindoles under benign reaction conditions, with CB600 demonstrating superior activity as a result of its elevated total basicity. This breakthrough achievement clarifies the critical role of basic biochars in Knoevenagel condensation-based reactions, highlighting their unprecedented utility for facilitating multicomponent synthesis of diverse heterocyclic compounds.

## Abstract 2

An efficient and sustainable route for the construction of diversified spirooxindoles using basic biochars as robust heterogeneous carbocatalysts.

## Graphical abstract

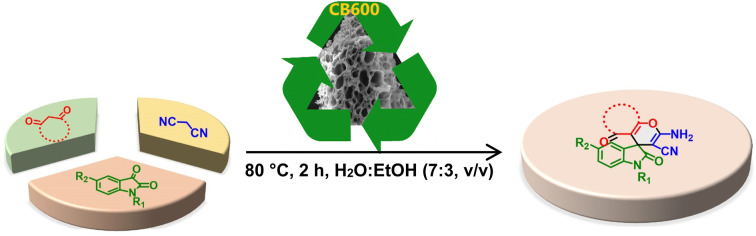

## 1 Introduction

In recent years, the pursuit of sustainable development has drawn significant attention to the utilization of renewable feedstocks as a viable alternative to conventional fossil-based resources. Biomass, derived from renewable feedstocks, has emerged as a feasible solution to traditional petrochemical-based materials due to its abundance, biodegradability, and minimal environmental footprint.^1–3^ The utilization of biomass in organic synthesis not only mitigates waste accumulation and reduces reliance on non-renewable resources but also adheres to green chemistry principles through the enhancement of atom economy and minimizing hazardous byproducts.^4,5^ Moreover, the ubiquitous nature and economic viability of biomass-derived materials makes them attractive for large-scale applications, particularly in catalysis, where cost efficiency is a critical factor.

In this context, biochars derived from biomass have gained significant attention as sustainable carbocatalysts, offering a renewable platform for a wide range of reactions involved in the catalytic conversion of biomass feedstocks into valuable chemicals.^6–10^ With its long-term stability, rich pore structure, and abundance of surface functional groups and inherent minerals, biochar also demonstrates exceptional potential as a flexible scaffold for constructing a range of functionalized carbon-based materials,^11^ a remediation agent targeting the removal of toxic contaminants,^12,13^ and an energy storage material.^14^ Given this broad spectrum of applications, biochar represents a promising substitute for conventional heterogeneous catalysts, which are associated with some known demerits such as high cost and environmental unfriendliness. Despite the advances in the chemistry and applications of biochar, its use in catalysis remains a burgeoning and fascinating theme, and the research in this field currently is confined to a limited investigation such as biodiesel production,^15–20^ tar removal or mitigation,^21,22^ syngas production,^23–26^ and biomass hydrolysis/bio-oil upgrading^27–29^ while its catalytic potential in organic transformations remains largely unexplored. In our recent study, the basic property potential of biochars was found to be significant (p*K*_b_ = 5.72–7.93) as evidenced by potentiometric titration, which varied according to the biochar feedstock.^30^ Notably, among the biochars evaluated, cow manure-derived biochar demonstrated the highest basicity, which enabled its effective application as a heterogeneous base catalyst in the synthesis of various heterocyclic compounds. These findings, particularly regarding cow manure-derived biochar, together with recent reports highlighting its catalytic applications,^31–35^ suggest that this biochar could be effectively utilized as a sustainable base catalyst in multicomponent reactions (MCRs), opening up a new avenue for its application in catalysis. Such potential not only emphasizes the versatility of cow manure-derived biochar in sustainable chemical processes but also motivates further exploration of its structure–property relationships to optimize catalytic performance.

Nowadays, MCRs are becoming flexible synthetic toolbox for the accelerated and proficient formation of structurally diverse and molecular entities and naturally comply with many of stringent requirements for ideal organic syntheses.^36–40^ In the MCR approach, the integration of multiple transformations within a single one-pot process using heterogeneous catalysts provides significant advantages over conventional linear-type synthesis, as they possess distinct features such as high atom economy, waste prevention, and low-cost separation and purification of products.^41^ Building upon these precedents and our interest in the synthesis of biologically significant heterocycles *via* a one-pot multicomponent approach,^42–44^ we sought to explore multicomponent reactions to prepare structurally diverse spirooxindoles by using biochar as a sustainable basic carbocatalyst, anticipating that its inherent basicity would effectively promote the reaction with high efficiency.

The synthesis of spirooxindoles is of particular importance to chemists due to their crucial significance in medicinal and pharmaceutical sciences.^45–48^ To date, some inspiring synthetic methods were given by many groups, such as transition metal-catalyzed reactions,^49^ multicomponent reactions,^50^ Heck reactions,^51^ intramolecular cyclizations, and intermolecular annulations,^52–54^ which can produce many structurally significant spirooxindole scaffolds. Despite the advances achieved in the chemistry of spirooxindoles, the use of expensive, non-sustainable and non-recyclable catalysts, along with strict reaction conditions and tedious work-up procedures, would result in low efficiency, limited scalability, and increased environmental impact. These challenges underscore the urgent need for more cost-effective and environmentally benign synthetic strategies and catalytic systems. In the present study, the multicomponent synthesis of spirooxindoles was chosen as a model reaction due to the structural complexity and pharmacological significance of these heterocyclic scaffolds. Moreover, this reaction pathway requires a basic catalyst, making it particularly suitable for evaluating the catalytic efficiency of cow manure-derived biochar under sustainable and environmentally benign conditions.

In light of the favorable characteristics of biochar as a safe carbocatalyst, herein, we wish to report on a new and efficient one-pot procedure for the direct synthesis of spirooxindoles utilizing biochar as a sustainable basic carbocatalysts. This approach not only leverages the inherent basicity, renewability, and recyclability of biochar, but also adheres to green chemistry principles by minimizing waste production and lowering the reliance on hazardous reagents. The developed catalytic system offers potential environmental, cost benefits and a practical route to structurally diverse spirooxindoles under mild reaction conditions, ensuring high efficiency, high product yields and ease of operation.

## 2 Experimental

### 2.1 General: biochar production

In the present work, biochars from different sources were used as heterogeneous catalysts toward spirooxindole production. Cow and sheep manure were collected from local livestock farms in Darab region (Fars Province, Iran). Licorice root pulp was obtained from a local herbal extraction factory in Darab. Municipal solid waste compost was collected from the Shiraz municipal composting facility. These materials were used after pretreatment:^30,55^ following collection, the feedstocks were air-dried for 48 hours, mechanically shredded using a high-speed grinder, and subsequently oven-dried at 105 °C for 24 hours. The dried biomass samples were then subjected to pyrolysis in an electric muffle furnace at target temperatures of 300 °C and 600 °C under limited oxygen conditions. The temperature was increased gradually from ambient to the target level at a heating rate of 5 °C per minute. Each pyrolysis process was maintained for 2 hours to ensure uniform thermal treatment. After cooling to room temperature, the resulting biochars were sieved through a 0.5 mm mesh to achieve uniform particle size. The biochars obtained from cow manure at 300 °C and 600 °C, sheep manure at 600 °C, licorice root pulp at 600 °C, and municipal compost at 600 °C were designated as CB300, CB600, SB600, LB600, and MB600, respectively.

#### 2.1.1 General procedure for synthesis of spirooxindoles

A mixture of isatin (1 mmol), malononitrile (1 mmol), 1,3-dicarbonyl compound (1 mmol) was discharged into a 25 mL round bottom flask containing 10 mg of CB600 in 3 mL of H_2_O : EtOH (7 : 3, v/v) as the reaction solvent. The reaction was stirred at 80 °C for 2 h until TLC analysis showed the completion of the reaction. After cooling to room temperature, the CB600 was separated from the reaction using a pad of Celite and the reaction mixture was then extracted with EtOAc (3 × 10 mL), and the organic layers were combined, dried over anhydrous Na_2_SO_4_, and concentrated under reduced pressure. The crude product was purified by recrystallization from ethanol to afford the pure spirooxindole derivatives. The products were confirmed by spectral data and physical characterization.

#### 2.1.2 Potentiometric titration of biochar samples

To evaluate the basicity constant of the biochars, 1 mg of each sample was dispersed in 1.0 mL of distilled water to obtain a stock suspension. For the potentiometric titration, 300 μL of the stock suspension was further diluted with 8 mL of distilled water containing 0.1 M KCl to maintain a constant ionic strength throughout the procedure. The resulting dispersion was titrated with incremental additions of 0.1 M HCl under continuous magnetic stirring. Following each addition of the titrant, the pH was measured using a calibrated pH meter to monitor the titration progress, and the next aliquot was introduced once the pH remained stable for approximately 20 seconds. The experimental data, representing pH as a function of titrant volume, were fitted using CurveExpert software to determine the pH at the midpoint of the equivalence point.

## 3 Results and discussion

**Fig. 1:**
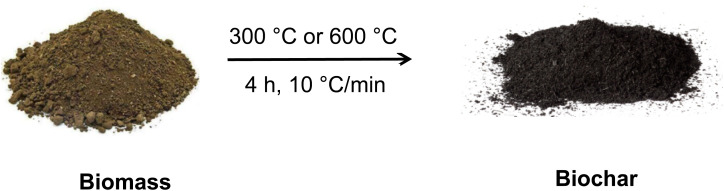
Slow-pyrolysis of biomasses for generation of biochars.

In the current study, biochars were derived from various sources such as animal dung (cow and sheep manure), plant waste (licorice pulp), and municipal compost. Then the biomass feedstocks underwent pretreatment, drying, grinding, and controlled pyrolysis under low oxygen circumstances at different temperatures to obtain biochars with uniform particle size (Fig. 1).

The as-synthesized biochars were characterized by Fourier transform infrared (FT-IR) spectroscopy, X-ray diffraction (XRD), scanning electron microscopy (SEM), and energy-dispersive X-ray spectroscopy (EDX) (see the SI). Following the initial characterization, a series of potentiometric titrations was conducted to quantitatively evaluate the basicity of the synthesized biochars. The resulting basicity constants for the samples are presented in Table 1.

**Table 1:** Basicity constant of biochars obtained by potentiometric method

Biochar	CB300	CB600	SB600	LB600	MB600
p*K*_b_	6.69	5.67	8.09	7.10	7.09

As evident from Table 1, CB600 exhibited the highest basicity and the representative data obtained from biochar titrations show a good correlation with the chemical characteristics of the biochars (see Table S1 in SI). The pH values together with the ash content presented in Table S1 support the outcomes of the titration experiments. The catalytic active sites in the biochars are associated with both the carbonaceous matrix and the alkaline mineral phases present. IR and XRD analyses confirm the presence of CaCO_3_, calcium hydroxyapatite, and Ca_3_(PO_4_)_2_ in CB600. These calcium-based minerals, which become more crystalline at higher pyrolysis temperatures (600 °C), are well known to act as basic catalytic sites.^56–58^ Theses alkaline mineral constituents not only increase the pH of the liquid-phase microenvironment but also provide catalytically active sites that facilitate spirooxindole formation. In relation to the catalytic activity of cow manure biochar, recently Qin *et al.* demonstrated that cow manure-derived biochar—particularly its capacity for catalyzing the degradation of 1,3-dichloropropene—is markedly influenced by pyrolysis temperature and moisture content, exhibiting a U-shaped dependency on these parameters.^35^ In another recent study, the application of cow manure-derived biochar significantly increased soil organic carbon storage, and reduced heavy metal uptake—effects attributed to its high surface area, well-distributed pore structure, and active functional sites that immobilize metal ions. Incorporating these findings alongside Qin *et al.*'s insights on the key role of pyrolysis temperature in modulating catalytic behavior underscores the value of systematically comparing our biochar samples with these well-characterized material.^59^ Incorporating these findings highlights the importance of systematically comparing our biochar samples with well-characterized materials. Building upon these insights and through benchmarking our samples against established data, we aim to identify optimal production parameters that enhance catalytic efficacy, thereby advancing the application of biochar in sustainable chemical processes. For instance, our biochar sample CB600 exhibits a higher specific surface area (84.7 m^2^ g^−1^) compared to previously reported cow manure biochars (*e.g.*, CBB600: 13.93 m^2^ g^−1^), while the pH of our biochar is higher than that of the previously reported samples and the ash content remains similar. Even compared to CB300, which has a reported specific surface area of 3.52 m^2^ g^−1^, CB300 demonstrates a substantially higher surface area, highlighting the superior textural properties of our biochar.^30,35^

Based on these insights and the pKb values and chemical characteristics of the biochars, the catalytic applications of these carbonaceous materials were investigated for the synthesis of spirooxindoles using isatins, malononitrile, and 1,3-dicarbonyl compounds as coupling partners. Initially, the reaction conditions were optimized by conducting a model reaction between isatin (1a) (1 mmol), malonitrile (2a) (1 mmol) and dimedone (3a) (1 mmol) in the presence of the biochars under different conditions. The results are summarized in Table 2. In the absence of the catalyst, the model reaction failed to yield the desired spirooxindole 4a at 80 °C even after 24 h, confirming that a catalyst is essential for the reaction to proceed. The use of CB300 (entry 2) significantly improved the yield to 57% within 4 hours, demonstrating the catalytic role of biochar. Increasing the pyrolysis temperature to 600 °C (CB600, entry 3) further enhanced the yield of the desired product 4a to 73%, likely due to increased porosity and a higher concentration of active sites.

**Table 2:** Model reaction and optimization^a^

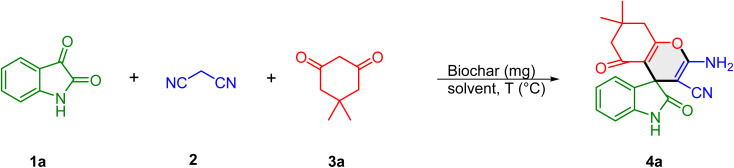					
Entry	Biochar (mg)	Solvent	*T* (°C)	Time (h)	Yield^b^ (%)
1	—	H_2_O	80	24	—
2	CB300 (10)	H_2_O	80	4	57
3	CB600 (10)	H_2_O	80	4	73
4	SB600 (10)	H_2_O	80	4	39
5	LB600 (10)	H_2_O	80	4	50
6	MB600 (10)	H_2_O	80	4	53
**7^c^**	**CB600 (10)**	**H**_**2**_ **O : EtOH (7 : 3, v/v)**	**80**	**2**	**86**
8	CB600 (10)	H_2_O : EtOH (1 : 1, v/v)	80	2	78
9	CB600 (10)	EtOH	Reflux	4	67
10	CB600 (10)	CHCl_3_	Reflux	8	44
11	CB600 (10)	THF	Reflux	8	52
12	CB600 (10)	CH_3_CN	Reflux	4	71
13	CB600 (10)	Solvent-free	80	12	<10
14	CB600 (10)	H_2_O : EtOH (7 : 3, v/v)	85	2	85
15	CB600 (10)	H_2_O : EtOH (7 : 3, v/v)	70	2	76
16	CB600 (10)	H_2_O : EtOH (7 : 3, v/v)	60	4	69
17	CB600 (10)	H_2_O : EtOH (7 : 3, v/v)	50	5	48
18	CB300 (10)	H_2_O : EtOH (7 : 3, v/v)	80	4	74
19	SB600 (10)	H_2_O : EtOH (7 : 3, v/v)	80	4	46
20	LB600 (10)	H_2_O : EtOH (7 : 3, v/v)	80	4	56
21	MB600 (10)	H_2_O : EtOH (7 : 3, v/v)	80	4	63
22	CB600 (7)	H_2_O : EtOH (7 : 3, v/v)	80	2	77
23	CB600 (15)	H_2_O : EtOH (7 : 3, v/v)	80	2	82

a Experimental conditions: isatin 1a (1 mmol), malonitrile 2 (1 mmol), and dimedone 3a (1 mmol), biochar (type indicated), and solvent (3 mL).

b Yield of pure isolated product.

c Bold value signifies the best reaction conditions.

Other biochar catalysts from different precursors were also examined under identical conditions. SB600 (entry 4) provided a lower yield of 39%, while LB600 (entry 5) and MB600 (entry 6) resulted in nearly identical yields of 50% and 53%, respectively. These results suggest that the physicochemical properties of biochar, especially their basicity, influenced by both precursor type and pyrolysis conditions, significantly affect catalytic efficiency. CB600 consistently outperformed other catalysts, establishing it as the most effective option (entry 7). To further enhance the reaction efficiency, the solvent system was modified. As the catalyst tended to agglomerate in water, a 7 : 3 mixture of water and ethanol was employed as the solvent. This modification significantly improved the reaction yield to 86%, likely due to enhanced dispersion of the catalyst. Changing the solvent ratio to 1 : 1 (entry 8) slightly decreased the yield to 78%, suggesting that the optimal ethanol proportion is necessary to maintain catalyst efficiency. When ethanol was used as the solvent under reflux (entry 9), the yield dropped to 67%. Other organic solvents such as CHCl_3_, THF, and CH_3_CN were also tested (entries 10–12) and performed moderately; however none of them exceeded the efficiency of the aqueous-ethanol system. When the reaction was conducted in the presence of CB600 under solvent-free conditions, an extremely low yield (<10%) of the product was obtained (entry 13). The influence of temperature on this transformation was subsequently examined. At 70 °C, the product was obtained in 76% yield within 2 h (entry 15), while decreasing the temperature to 60 °C significantly lowered the yield to 69% under the same reaction time (entry 16). A further decrease to 50 °C resulted in only 48% yield even after 5 h, demonstrating the strong dependence of the reaction efficiency on temperature (entry 17). On the other hand, increasing the temperature to 85 °C, provided the final product 4a in 85% yield within 2 h (entry 14). These findings indicate that the optimal condition is 80 °C (86% yield, 2 h), as raising the temperature beyond this point does not significantly improve the yield. Further optimizations involved testing different carbocatalysts in the optimized H_2_O : EtOH (7 : 3) medium (entries 18–21), where CB300 showed moderate efficiency, while SB600, LB600, and MB600 exhibited lower catalytic performance, reaffirming CB600 as the superior catalyst. Finally, the effect of catalyst loading was examined. CB600 showed poor performance, providing 77% isolated yield when the catalyst loading was decreased to 7 mg (entry 22). A further increase in catalyst loading to 15 mg resulted in no significant improvement beyond the optimal yield of 86%, indicating that 10 mg is the optimal loading for maximum efficiency (entry 23). Having developed an effective MCR protocol, as optimized on 4a, we looked to examine the scope with other substrates (Table 3). Initially, isatin 1a and malononitrile 2 were set up to undergo reactions with a variety of β-dicarbonyls 3. As depicted in Table 2, various β-dicarbonyls, such as dimedone, barbituric acid, thiobarbituric acid, 4-hydroxycoumarin, pyrazolone, and 2,3-dihydro-5*H*-[1,3]thiazolo[3,2-*a*]pyrimidine-5,7(6*H*)-dione can take part in the reactions, delivering the target products 4a–f in synthetically useful yields (Table 3, entries 1–6). The substrate scope of the CB600-catalyzed multicomponent reaction was further investigated using various isatin derivatives. The reaction of dimedone and malonitrile with *N*-benzyl isatins and 5-nitroisatin demonstrated consistently high yields ranging from 90% to 94%, indicating that substituted isatins are compatible with this MCR process to give the spiro compounds in satisfactory yields (entries 7–9). When barbituric acid, 1,3-dimethylbarbituric acid, and 4-hydroxycoumarin were employed as substrates to react with malonitrile and 5-nitroisatin, the corresponding spirooxindoles 4j–4l were obtained in high yields (entries 10–12).

**Table 3:** CB600-catalyzed synthesis of spirooxindoles^a^

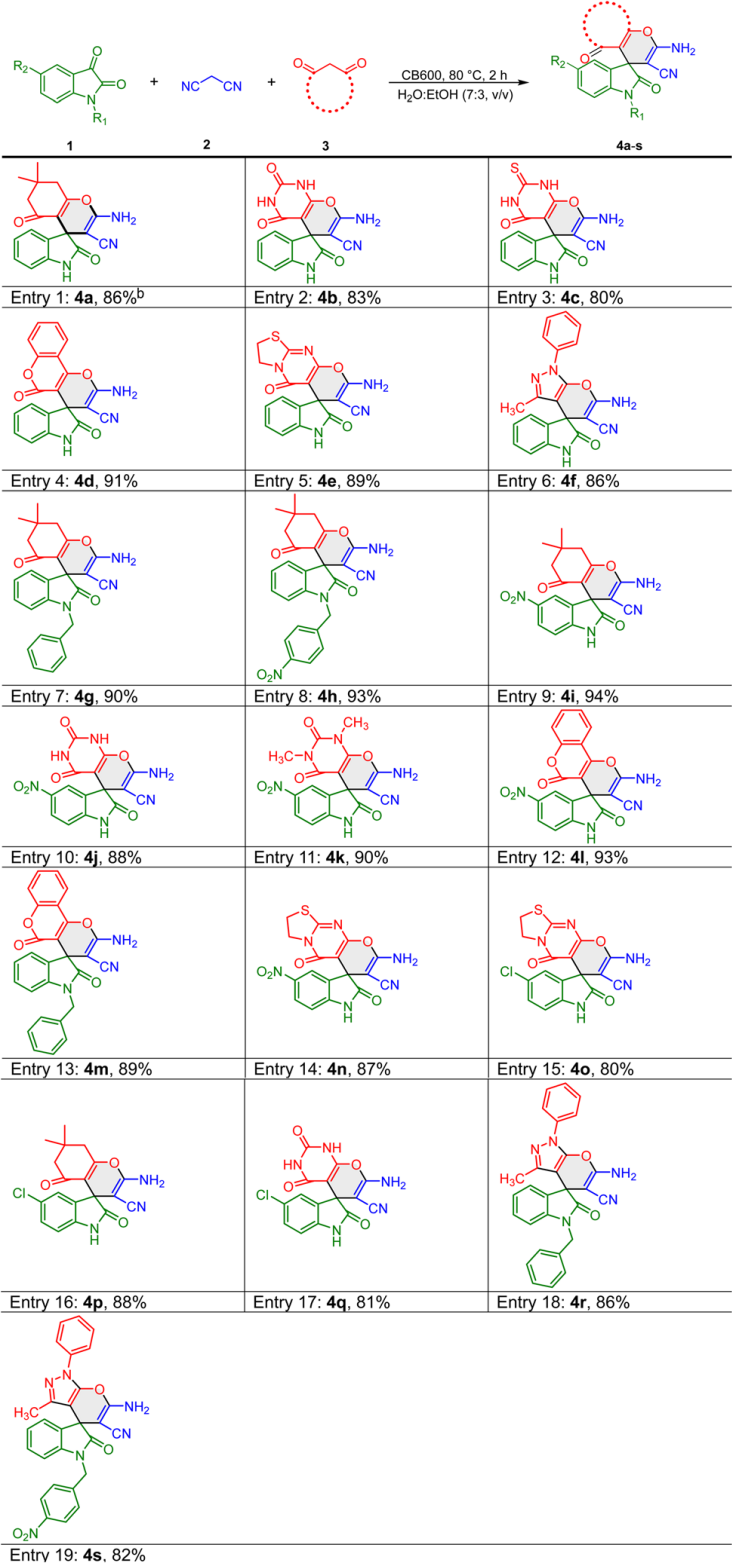

a Experimental conditions: isatins 1 (1 mmol), malonitrile 2 (1 mmol), and β-dicarbonyls 3 (1 mmol), CB600 (10 mg), and H_2_O : EtOH (7 : 3, v/v) (3 mL).

b Yield of pure isolated product.

4-Hydroxycoumarin underwent a smooth coupling reaction with malonitrile and *N*-benzylisatin to afford the corresponding spirooxindole product 4m in high yield (89%, entry 13). Further, it was found that the reaction of 2,3-dihydro-5*H*-[1,3]thiazolo[3,2-*a*]pyrimidine-5,7(6*H*)-dione with malonitrile and isatin bearing electron-withdrawing substituents such as 5-nitro and 5-chloro groups on isatin resulted in the formation of spirooxindoles 4n–p in 87% and 80% yields, respectively (entries 14–15). Similarly, the reaction of 5-chloroisatin with malonitrile and β-dicarbonyls such as dimedone and barbituric acid furnished the desired products 4p and 4q in 88% and 81% yields, respectively (entries 16 and 17). Much to our satisfaction, the optimized conditions were mild enough to enable the reaction between *N*-benzylisatin substrates and 3-methyl-1-phenyl-5-pyrazolone, affording the corresponding products 4r and 4s (entries 18 and 19, 82 and 86% yield).

**Fig. 2:**
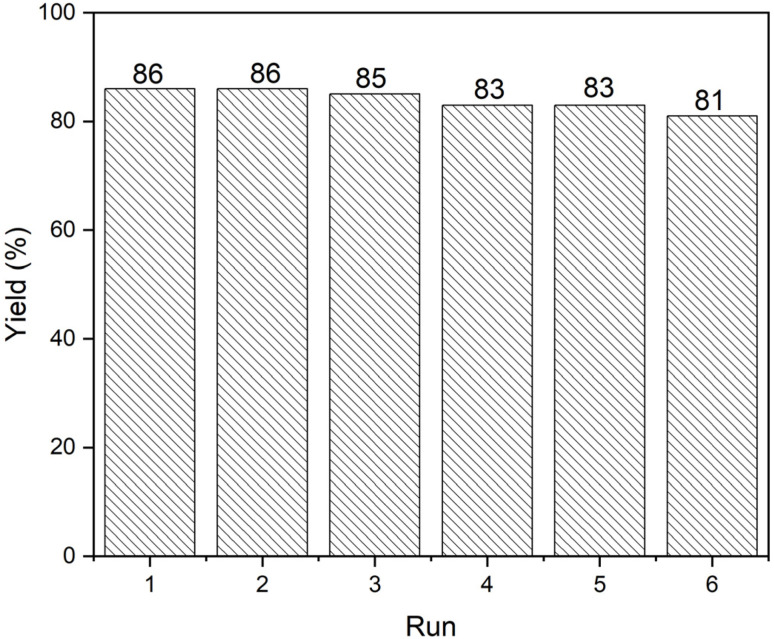
Recyclability and reusability data of CB600 in the synthesis of 4a: conditions: isatin (5 mmol), malonitrile (5 mmol), dimedone (5 mmol), CB600 (50 mg), and H_2_O : EtOH (7 : 3, v/v) (10 mL).

As catalyst recovery is vital in sustainable and cost-effective chemical processes, the recyclability and reusability of the catalyst were evaluated through its application in successive cycles of the model reaction. Upon completion of the reaction for the synthesis of compound 4a, the carbocatalyst CB600 was recovered by filtration. The separated catalyst was subsequently washed multiple times with deionized water and ethanol, and then dried at 60 °C for 12 hours prior to reuse. The performance of the recovered catalyst was subsequently evaluated over six consecutive reaction cycles, with the corresponding product yields presented in Fig. 2.

The results indicated that across all six reaction cycles, the yield of compound 4a exhibited only a slight decline with each successive reuse of the catalyst. To provide a more comprehensive evaluation of the catalytic performance of CB600, its turnover numbers (TONs) and turnover frequency (TOF) were calculated for the model reaction.^60^ Under the given conditions, the overall turnover numbers (TONs) across six cycles were found to be 504 mmol g^−1^. This heterogeneous catalytic system also exhibited turnover frequencies (TOF) of approximately 0.42 h^−1^, indicating a high and acceptable level of performance.^7^ Green chemistry metrics are employed to quantify the efficiency and environmental impact of chemical processes, enabling the assessment and comparison of performance improvements over time.^61^ To evaluate the environmental sustainability of the catalytic system, some important key green metrics such as the environmental factor (E-factor)^62^ and atom economy^63^ were calculated for the model reaction, as summarized in Table 4.

**Table 4:** Green chemistry metrics of the catalytic system

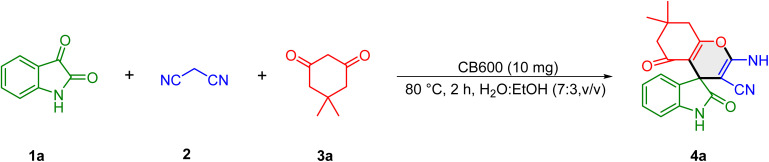		
Factors	Value	
E-factor	0.22 kg kg^−1^	
% atom economy	95%	
Reaction mass efficiency (RME)	82%	
Process mass intensity (PMI)	1.23	Ideal value of PMI = E-factor + 1 (=1.22)
E-score	84	>75: excellent
		>50: acceptable
		<50: inadequate

Using the CB600-catalyzed reaction between isatin, malonitrile, and dimedone, as an example, the E-factor of the process was 0.22 kg kg^−1^. For this catalytic system, the atom economy which indicates the efficiency of a chemical process and sustainability, was determined to be 95%, which was significantly high, showcasing the system's remarkable alignment with green chemistry principles. Reaction Mass Efficiency (RME)^64^ and Process Mass Intensity (PMI)^65^ are essential quantitative metrics frequently utilized in evaluating environmental sustainability, offering a more realistic measure of a reaction's “greenness”. Under the optimized conditions, the RME of the present catalytic system was calculated to be 82%, reflecting the efficiency and environmentally favorable nature of the process. An optimal process mass intensity (PMI) value of 1.23 was also achieved with this catalytic system, demonstrating its proximity to the theoretical ideal value of 1.22. Ecoscale,^66^ an evaluation framework based on both economic and ecological principles, functioning as a vital green chemistry metric for evaluating the sustainability and environmental impact of chemical reactions. The eco-scale of the catalytic system was calculated to be 84, indicating a high level of environmental compatibility of the process, suggesting that the process achieves an optimal balance between economic and ecological considerations.

**Fig. 3:**
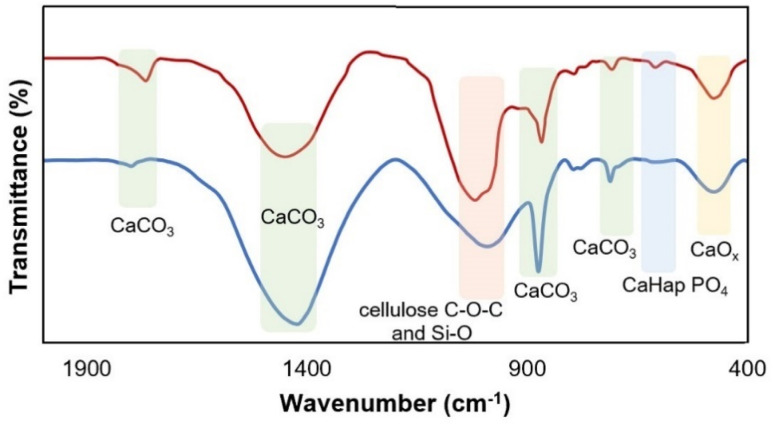
FT-IR spectra of fresh (blue) and six-times reused CB600 (red).

The characterization of the used catalyst has also been carried out to study the changes of the catalyst during the reaction. The FT-IR spectra of the CB600 sample before and after the reaction were displayed in Fig. 3. No remarkable change could be noted in the peak pattern of the reused CB600, showing that the active functional groups remained intact in the recycled catalyst without notable structural modifications.

**Fig. 4:**
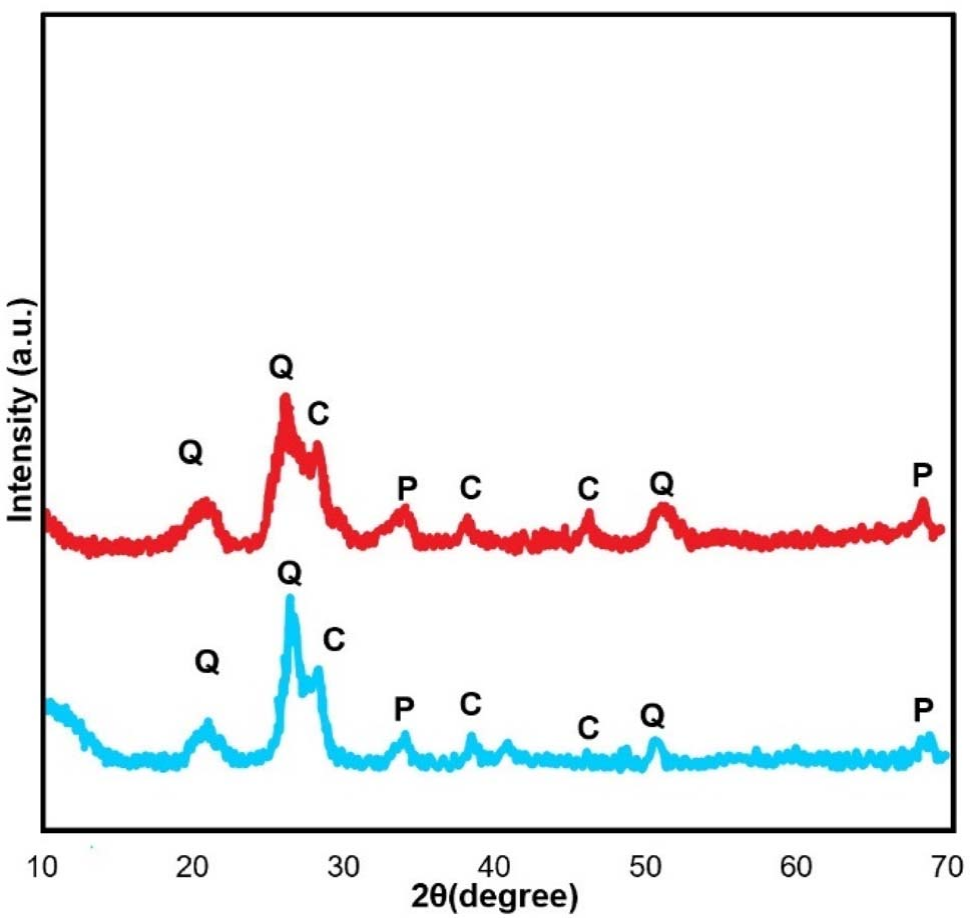
XRD patterns of fresh (blue) and six-times reused CB600 (red).

XRD analysis was also performed to monitor the fate of the catalyst in the model reaction after the sixth run (Fig. 4). The reused catalyst exhibited well-resolved diffraction peaks, suggesting that the structure could be well preserved after multiple reaction cycles.

**Fig. 5:**
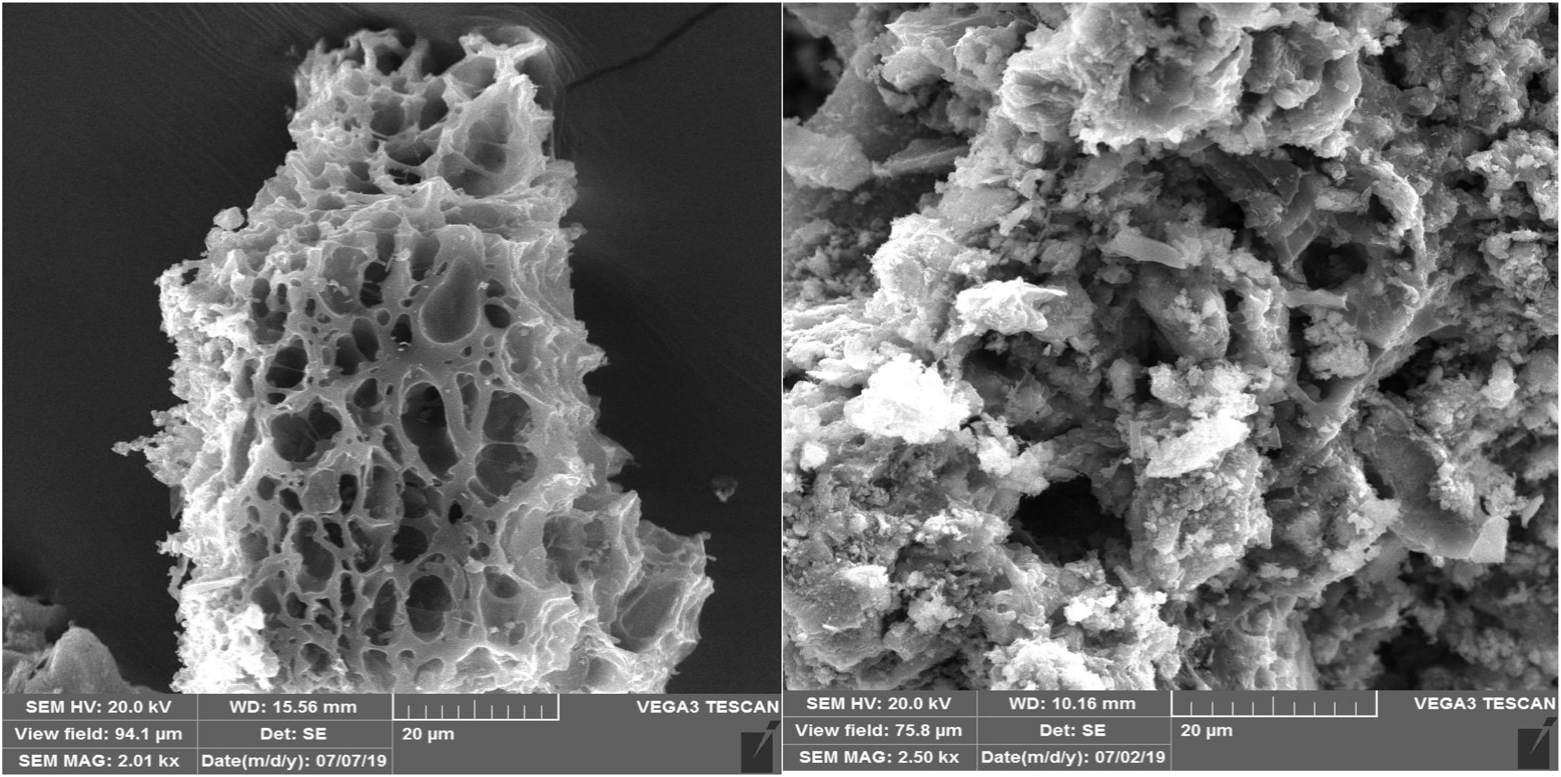
SEM images of fresh (left) and six-times reused CB600 (right).

Scanning electron microscopy (SEM) was also conducted to investigate the morphological evolution of the catalyst surface during the course of the reaction (Fig. 5).

While the SEM image of fresh CB600 exhibited a more developed and ordered porous network with a honeycomb-like structure, the reused CB600 after six catalytic cycles displayed a markedly altered morphology. The increase in pore uniformity in the fresh sample highlights the significant impact of elevated pyrolysis temperature (600 °C) on enhancing porosity. In contrast, the SEM image of the reused sample revealed a denser surface, collapse of the porous framework, and the presence of irregular aggregated particles, suggesting surface fouling, pore blockage, and partial structural degradation due to repeated catalytic use. As shown in Fig. 5, the partial collapse of the biochar structure after six successive reaction cycles does not negate the contribution of surface area and porous structure to the catalytic performance. Rather, this observation highlights the involvement of another important factor, namely the presence of basic mineral components within the biochar matrix, which also play a decisive role in sustaining the catalytic activity.

**Fig. 6:**
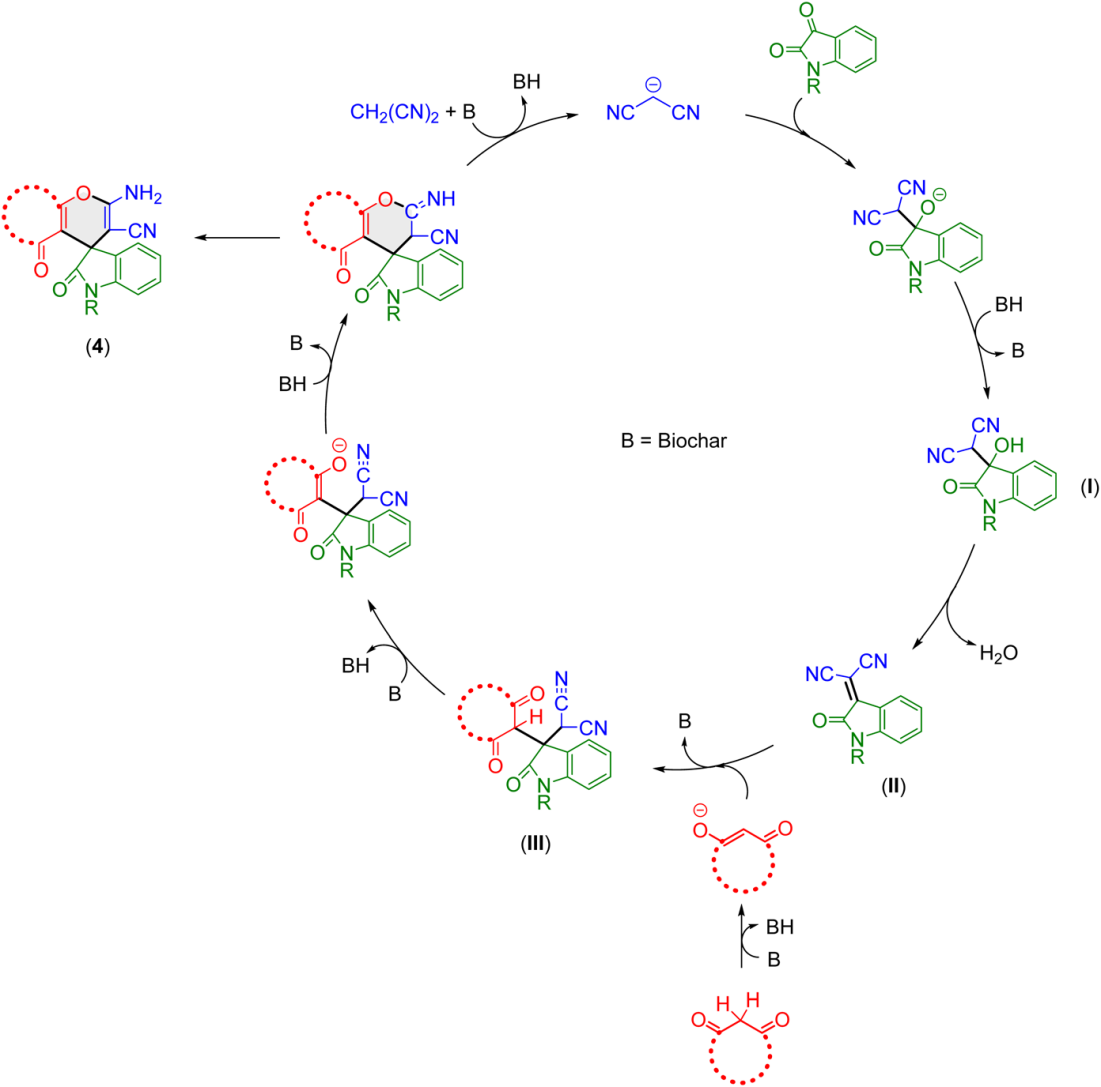
Possible reaction pathway for the synthesis of spirooxindoles in the presence of CB600.

Based on previous literature reports,^67,68^ and our experimental results, the mechanistic pathway for the synthesis of spirooxindole derivatives 4a–s catalyzed by CB600 is represented in Fig. 6. The mechanism underlying spirooxindole synthesis can be described as a sequence of consecutive steps, comprising a Knoevenagel condensation, a Michael addition, and a subsequent intramolecular cyclization, which together contribute to the formation of the final product. The reaction proceeds through the initial formation of a dicyanomethanide anion by abstracting a methylene proton from malononitrile, a process that is facilitated by biochar.

The dicyanomethanide anion then underwent a nucleophilic reaction on isatin, giving rise to intermediate (I), which upon dehydration, yielded the Knoevenagel product (II). Next, the Michael-type addition of β-dicarbonyls (which were also activated by the catalyst) to the Knoevenagel product (II) result in the insitu formation of the Michael adduct (III), which subsequently undergoes an intramolecular nucleophilic cyclization, leading to the formation of the desired spirooxindole derivative 4. Supporting evidence for the biochar-catalyzed synthesis of spirooxindoles was provided by an independent reaction of 2-oxo-2,3-dihydroindolylidenemalononitrile (II) with dimedone in the presence of CB600, which afforded the desired spirooxindole 4a in a yield of 86%. 2-Oxo-2,3-dihydroindolylidenemalononitrile was synthesized through a Knoevenagel condensation of malononitrile and isatin employing CB600 in H_2_O : EtOH (7 : 3, v/v). In this reaction, an orchid purple precipitate was isolated which tentatively identified as 2-oxo-2,3-dihydroindolylidenemalononitrile. The structure of the precipitated compound was confirmed by its ^1^H and ^13^C NMR spectra recorded in DMSO-d_6_ (SI). The obtained results confirm that the intermediate (II) is formed during the course of the present reaction.

## 4 Conclusion

In the present study, we have prepared a series of nanobiochars *via* pyrolysis-carbonization of various manures and organic waste materials. Among the nanobiochars, cow manure biochar obtained at 600 °C (CB600) exhibited the highest basicity, as confirmed by potentiometric titration and chemical characterization techniques. This enhanced basic nature directly translated into superior catalytic performance in the one-pot three-component synthesis of multifunctionalized spirooxindoles under mild and sustainable conditions. To provide a more comprehensive assessment of the catalytic potential of CB600, several key green chemistry metrics were calculated. The results demonstrated excellent performance across all metrics, highlighting the process's strong environmental compatibility and indicating an optimal balance between economic efficiency and ecological sustainability. These findings not only reinforce the value of nanobiochars as green, and basic carbocatalysts, but also underscore their potential as sustainable alternatives to conventional catalysts, offering enhanced performance while minimizing environmental impact. This work paves the way for greener and more cost-effective synthetic strategies for multicomponent synthesis of structurally diverse heterocycles in organic chemistry.

## Author contributions

Ali Khoy: investigation, methodology, formal analysis, data curation, software. Dariush Khalili: conceptualization, formal analysis, data curation, resources, software, writing – review & editing, supervision; Hamid Reza Boostani: investigation, data curation, software, formal analysis.

## Conflicts of interest

There are no conflicts to declare.

## Supplementary Material

RA-015-D5RA05293A-s001

## Data Availability

All data included in this study are available upon request by contact with the corresponding author (*i.e.* Dr Dariush Khalili, email: khalili@shirazu.ac.ir). The data supporting this article have been included as part of the SI. Supplementary information: chemical and instrumental details, characterization data for biochars, calculation of green chemistry metrics, and ^1^H NMR and ^13^C NMR spectra for all synthesized compounds (DOCX). See DOI: https://doi.org/10.1039/d5ra05293a.
