# Supplementary material for: Biochar: a robust and renewable basic carbocatalyst for sustainable synthesis of diversified spirooxindoles
Source: RSC Adv. 2025 Oct 3;15(44):36720–30. doi: 10.1039/d5ra05293a (PMC12495400; doi:10.1039/d5ra05293a)
Supplement: RA-015-D5RA05293A-s001 [file RA-015-D5RA05293A-s001.pdf]

Supplementary Information (SI) for RSC Advances.
This journal is © The Royal Society of Chemistry 2025

Biochar: a robust and renewable basic carbocatalyst for sustainable synthesis of diversified spiro-oxindoles

Ali Khoy,[1] Dariush Khalili,[1]* and Hamid Reza Boostani[2]

Corresponding author: Dariush Khalili

[1]*Department of Chemistry, College of Sciences, Shiraz University, Shiraz 71467-13565, Iran*

[2]*Department of Soil and Water engineering, College of Agriculture and Natural Resources of Darab, Shiraz University, Darab, Iran.*

Table of content

Experimental section……………………………………………………...………………………S2

Characterization of the biochars…………………………………………………………………S2

Calculation of green chemistry metrics…………………………………………………………..S9

Characterization of the products…………………………………..……..……………………..S12

References………………………………………………………………..…………………...S22

Copy of FT-IR, ^{1}H-NMR and ^{13}C-NMR of products…………………..…………………………....…S25

Experimental section

Chemical and instrument

All chemicals were purchased from Merck, Acros, and Sigma-Aldrich and used without further purification. The power and oscillation of the used ultrasonic device in catalyst synthesis was 120 (V) and 40 (kHz) and the final catalyst was characterized as follows: The fourier transform infrared spectrometer (FT-IR) model Shimadzu FT-IR 8300 was applied to FT-IR measurement using KBr pellet in the range of 400 to 4000 wavenumbers/cm^{-1}. Also, the X-ray diffraction (XRD) patterns were recorded by a GNR (Italy) XRD explorer X-ray diffractometer using CuKα radiation (λ = 1.54178 Å) with a 2θ scan range of 10° to 70°. Moreover, the presence of elements in the biochar samples was proved by energy dispersive X-ray spectrometry (EDX) attached to a Philips scanning electron microscope (SEM). N_2 adsorption/desorption isotherms were measured on a BELSORP MAX G, BEL Japan at liquid nitrogen temperature. The reaction progress has been checked by thin layer chromatography (TLC). The final products characterized by melting points in open capillary tubes were determined with a Büchi B-545 melting point apparatus, and nuclear magnetic resonance (NMR) spectroscopy using Devices Brucker DPX-400 spectrometer that work for ^{13}C at 101 MHz and for ^{1}H at 400 MHz and a spectrometer Brucker DPX-300 that work for ^{13}C at 75 MHz and ^{1}H at 300 Hz in pure deuterated dimethyl sulfoxide (DMSO-d_6) and deuterated chloroform ($CDCl_3$).

Characterization of the biochars

The structural properties of the biochars were well characterized by Fourier transform infrared spectroscopy (FT-IR), scanning electron microscopy (SEM), energy-dispersive X-ray spectroscopy (EDX), and powder X-ray diffraction (XRD). FT-IR spectra (Figure S1) revealed that the CB300 displayed a vibration band at 470 cm^{-1}, which is indicative of the typical Ca-O bond. The CB300 sample showed a peak at 619 cm^{-1}, which was attributed to the phosphate functional groups in calcium hydroxyapatite. The absorbance peaks around 712, 873, 1427 and 1810 cm^{-1} could be assigned to the calcium carbonate[1]. The broad peak at around 1103-1136 cm^{-1} correspond to cellulose functionalities (1016 cm-

^{1})[2] and Si-O bond (1117 cm^{-1}). In FT-IR spectra, the CB600 sample displayed significantly absorption bands with greater intensity, corresponding to cellulose functionalities, as well as phosphate groups. In accordance with previous studies[3], the sample annealed at 600 °C (CB600) demonstrated a progressive increase in the intensity of $CaCO_3$ peaks. This trend is primarily attributed to the elevated formation of crystalline calcium carbonate as the charring temperature increases.

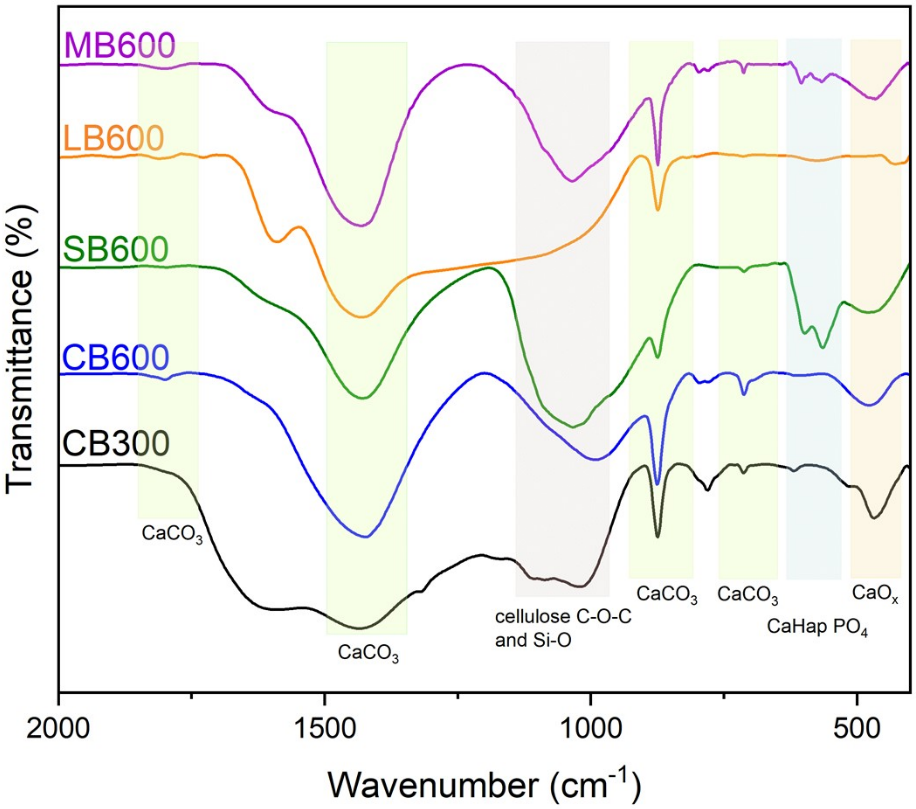

Fig. S1. FTIR spectra of the biochar samples: cow manure biochars produced at 300 °C (CB300), 600 °C (CB600), sheep manure biochar at 600 °C (SB600), licorice root pulp biochars at 600 °C (LB600), and municipal compost biochars at 600 °C (MB600).

The crystal structure of the biochars was also characterized by X-ray diffraction (XRD) as shown in Figure S2. The XRD patterns of the CB300 and CB600 exhibited well-resolved

diffraction peaks at around 2θ = 21.2°, 26.5° and 51.7° indicative of the quartz (SiO_2) crystalline [4].

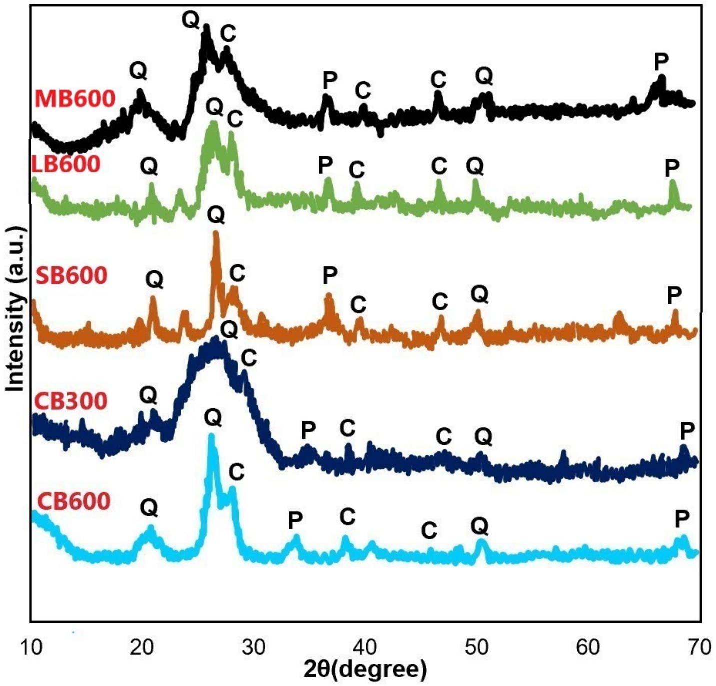

Fig. S2. Comparison of the XRD patterns of the prepared biochars. Q, C, and P represents features of quartz, calcite and calcium phosphate [Reprinted in part with permission from: Khalili, D.; Ramjerdi, A. A.; Boostani, H. R.; Ghaderi, A.. Biochar: a high performance and renewable basic carbocatalyst for facilitating room temperature synthesis of 4*H*-benzo[*h*]chromene and pyranopyrazoles in water. *Biochar* **2024**, *6*:6. Copyright © 2024 Springer Nature]

The presence of quartz provides clear evidence that the original feedstocks were rich in silicon, a conclusion further corroborated by the Si-O-Si stretching vibration band observed in the FT-IR spectra (see Fig. S1). The presence of three additional peaks at 2θ = ≈ 28°, 39.5° and ≈ 47° confirms the identification of calcite ($CaCO_3$) in biochars produced at two different temperatures [5]. The peaks around 2θ = 37° and 68°, likely

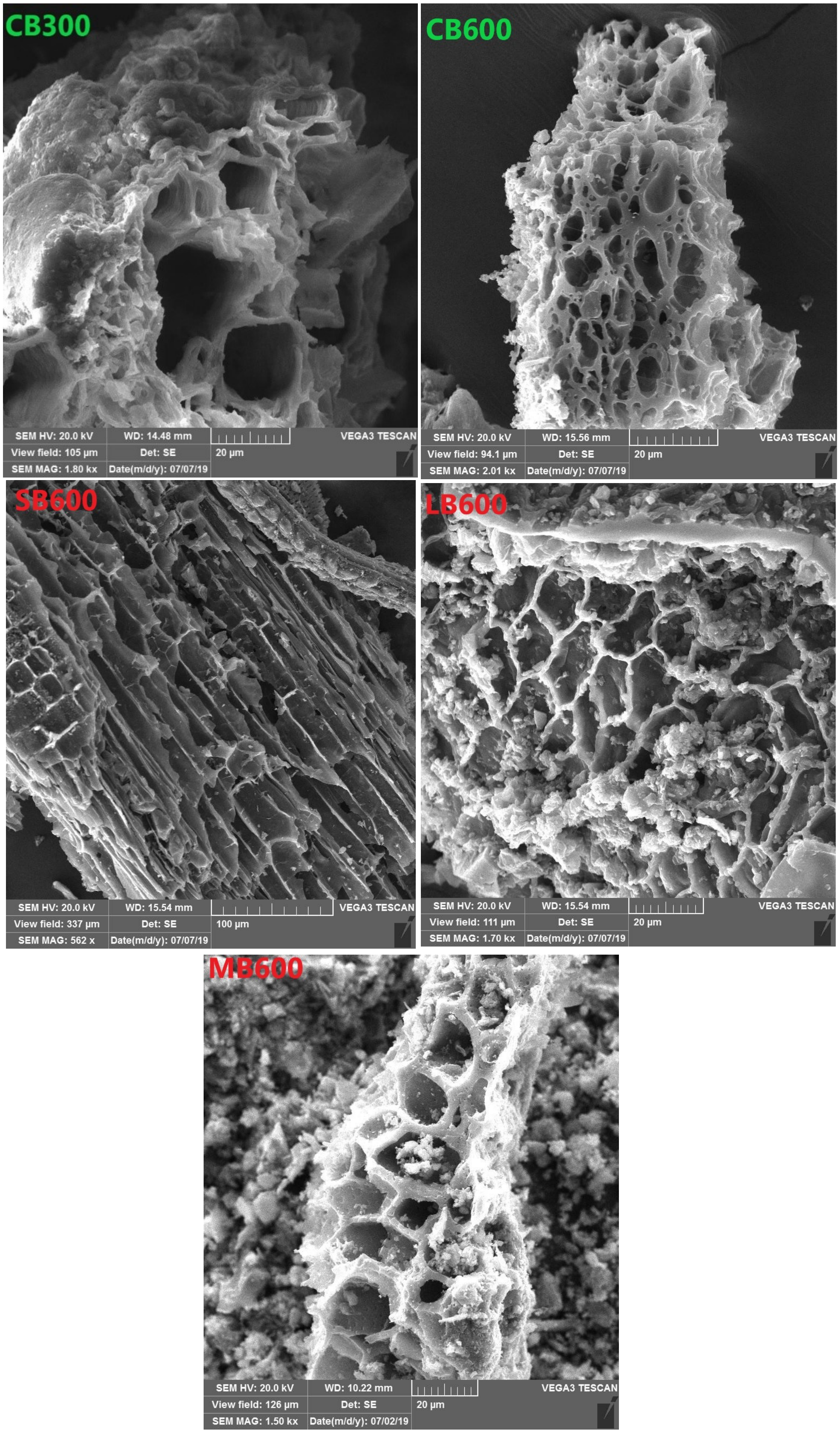
CB300
SEM HV: 20.0 kV
WD: 14.48 mm
VEGA3 TESCAN
View field: 105 µm
Det: SE
20 µm
SEM MAG: 1.80 kx
Date(m/d/y): 07/07/19
CB600
SEM HV: 20.0 kV
WD: 15.56 mm
VEGA3 TESCAN
View field: 94.1 µm
Det: SE
20 µm
SEM MAG: 2.01 kx
Date(m/d/y): 07/07/19
SB600
SEM HV: 20.0 kV
WD: 15.54 mm
VEGA3 TESCAN
View field: 337 µm
Det: SE
100 µm
SEM MAG: 562 x
Date(m/d/y): 07/07/19
LB600
SEM HV: 20.0 kV
WD: 15.54 mm
VEGA3 TESCAN
View field: 111 µm
Det: SE
20 µm
SEM MAG: 1.70 kx
Date(m/d/y): 07/07/19
MB600
SEM HV: 20.0 kV
WD: 10.22 mm
VEGA3 TESCAN
View field: 126 µm
Det: SE
20 µm
SEM MAG: 1.50 kx
Date(m/d/y): 07/02/19

Fig. S3. SEM images of CB300, CB600, SB600, LB600 and MB600

originate from the presence of $Ca_3(PO_4)_2$ which is consistent with the literature[6].

The SEM images of different biochars are shown in Fig. S3. The SEM image of CB300, captured at a magnification of approximately 1800×, revealed a relatively compact structure with irregular and sponge-like porous structure with visible and disordered pores. This morphology confirms the incomplete carbonization and the presence of residual organic material, characteristic of biochars produced at lower pyrolysis temperatures.

In contrast, the image of CB600, taken at a higher magnification of around 2000×, exhibited a more developed and ordered porous network with honeycomb-like structure. The increase in pore uniformity and density highlights the significant impact of elevated pyrolysis temperature (600 °C) on enhancing porosity through the release of volatile matter and the restructuring of the carbon matrix.

The SEM image of SB600 revealed a distinctly fibrous and layered morphology, characterized by elongated, compact structures with limited visible macroporosity. Unlike the more porous biochars derived from plant-based feedstocks, SB600 exhibited a denser and more ordered surface texture, likely reflecting the intrinsic structural composition and higher ash content of sheep manure (see Table S1).

The SEM image of LB600 displays a well-developed network of interconnected, tubular macropores. These pores appear to be embedded within an irregular matrix, with remnants of the original plant cell wall architecture still discernible. The biochar surface shows extensive porosity, likely a result of the fibrous and lignocellulosic nature of licorice root pulp, which facilitates structural decomposition and pore formation during pyrolysis. The observed macropores are closely associated with the walls of biochar particles, suggesting partial preservation of vascular tissue from the plant feedstock. The SEM image of MB600 reveals a heterogeneous and sponge-like morphology, characterized by irregularly shaped macropores interspersed with compact regions and surface-bound particulate residues. The irregularity in pore size and distribution reflects the complexity

of the feedstock, which typically comprises a mixture of plant debris, food waste, and inorganic matter.

EDX analysis of biochars CB300, CB600, SB600, LB600, and MB600 revealed rich amount of mineral elements (Fig. S4).

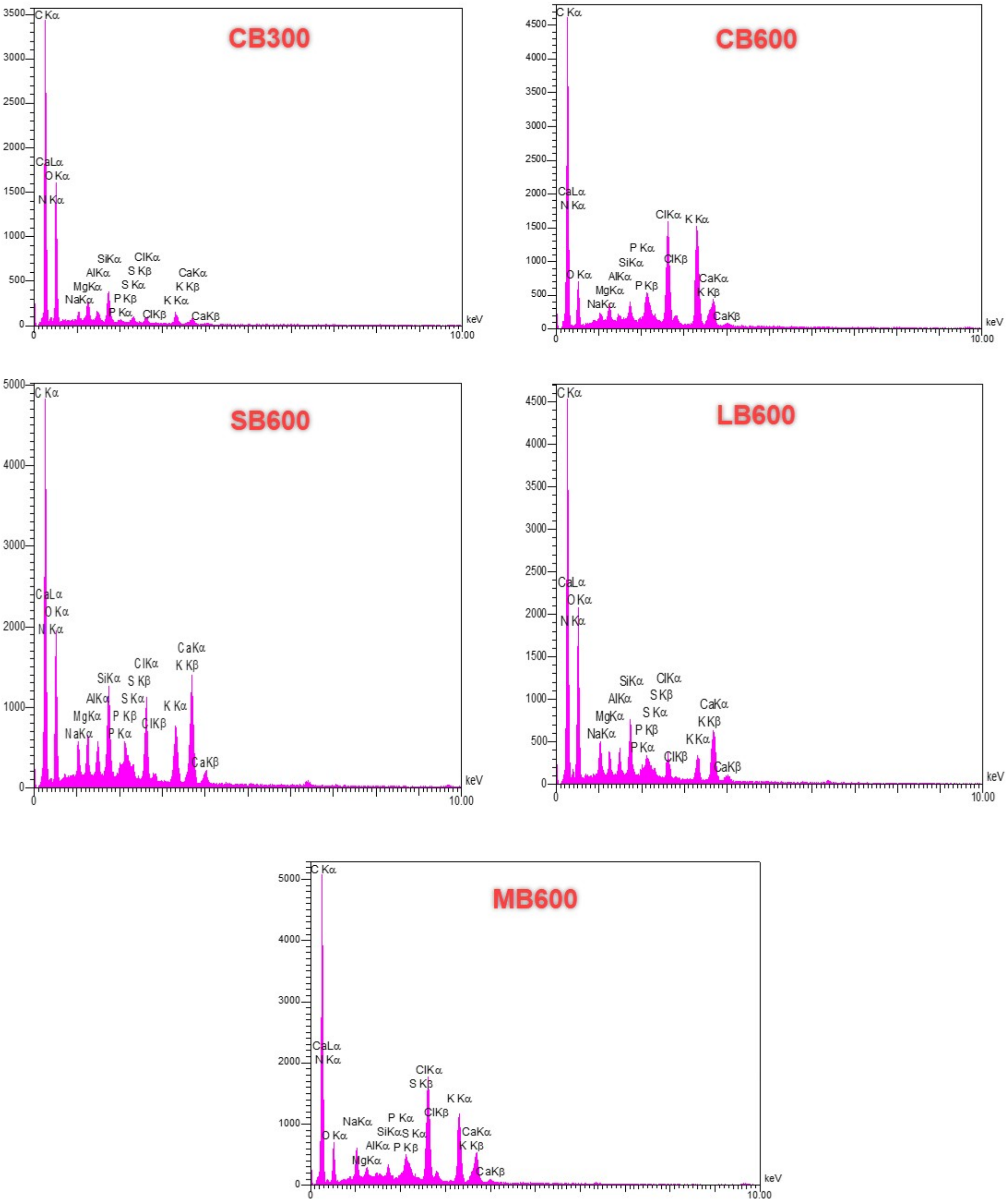

Fig. S4. EDX analysis of all produced biochars.

The chemical characteristics of the prepared biochars were also studied using established laboratory methods.

Table S1. chemical properties of the biochars

Property	CB300	CB600	SB600	LB600	MB600
H:C molar ratio	0.55	0.15	0.31	0.26	0.09
pH	10.49	11.70	10.19	9.12	10.70
Carbon (%)	59.1	55.6	50.23	69.4	53.29
Hydrogen (%)	2.7	0.7	1.29	1.51	0.4
Nitrogen (%)	3.26	2.19	2.70	2.54	2.98
Ash content (%)	27.21	49.19	54.01	23.65	42.31

CB300 = cow manure biochar produced at 300 °C; CB600 = cow manure biochar produced at 600 °C; SB600 = sheep manure biochar produced at 600 °C; LB600 = licorice root pulp biochar produced at 600 °C; MB600 = municipal compost biochar at 600 °C.

The pH values were determined using a suspension prepared with a solid-to-distilled water ratio of 1:10. The elemental composition, including total carbon (C), hydrogen (H), and nitrogen (N) contents, was analyzed using a CHN analyzer. As presented in Table S1, all of the prepared biochars exhibited a hydrogen-to-carbon (H:C) molar ratio of 0.6 or lower and a total carbon content of 50% or higher, indicating that they were sufficiently pyrolyzed to meet the criteria for classification as biochars. (According to the EBC and International Biochar Initiative (IBI)) (Comparison of European Biochar Certificate Version 4. 8 and IBI Biochar Standards Version 2. 0 European Biochar Certificate first publication March 2012). The hydrogen-to-carbon (H:C) molar ratio of the biochars also serves as an indicator of the extent of carbonization[7]. Lower H:C molar ratios are associated with a higher degree of aromatic condensation and an advanced stage of carbonization. Among the biochars, CB600 and MB600 had the lowest H:C mole ratio, reflecting a higher degree of aromatic condensation and carbonization. A comparison between CB300 and CB600 clearly demonstrates that increasing the pyrolysis temperature from 300 °C to 600 °C leads to a corresponding enhancement in the degree of carbonization. A comparison of the pH values between CB300 and CB600 indicates that an increase in charring

temperature from 300 °C to 600 °C results in a corresponding elevation in pH from 10.49 to 11.70. This increase in pH is attributed to the pyrolysis-induced formation of crystalline calcium carbonate, higher ash content, and the degradation of surface acidic functional groups[8]. These findings are further corroborated by the enhanced intensity of $CaCO_3$ peaks observed in the IR spectrum of CB600 (see Fig. S1). Overall, the biochars exhibited alkaline pH values, ranging from 9.38 to 11.51. Among them, CB600 recorded the highest pH (11.70), whereas LB600 showed the lowest (9.12). The ash content of the biochars varied significantly depending on both the feedstock type and the pyrolysis temperature, as presented in Table S1. The variation in ash content across different feedstocks at the same pyrolysis temperature (600 °C) highlights the influence of raw material composition on the final properties of biochar. Manure-based biochars (CB600, SB600, MB600) generally showed higher ash contents compared to plant-based biochar (LB600), due to the naturally higher mineral content in animal waste[9].

Calculation of green chemistry metrics

In order to assess the environmental sustainability of our catalytic system, important green chemistry metrics such as the environmental factor (E-factor), atom economy, reaction mass efficiency (RME), process mass intensity (PMI) and eco-score (scale) were scrutinized. Taking the BCN-catalyzed reaction involving phenylhydrazine, benzaldehyde, and ethyl acetoacetate as a model reaction, these metrics were obtained as follows:

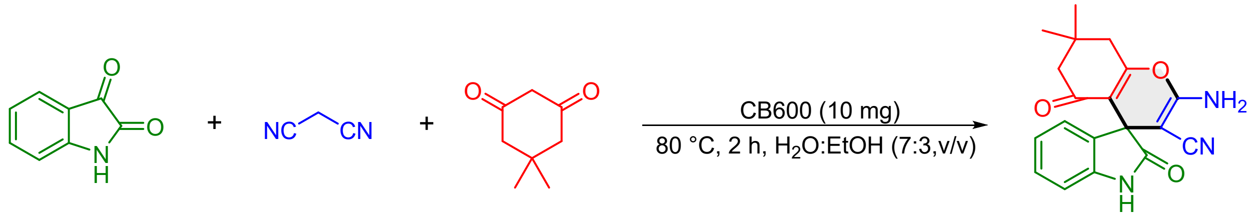

1a (1mmol) **2** (1mmol) **3a** (1mmol) **4a** (86%)

**Compound code**	**1a**	**2**	**3**	**4a**
M.W. (g/mol)	147	66	140	335
M.W. (mg)	147	66	140	335

The total mass of reactants = 353

Obtained product = 335 × 0.86 = 288.1

Environmental factor (E-factor):

E-factor = Amount of waste /Amount of product

The Amount of waste = (total mass of raw materials - the total mass of product)

The Amount of waste = (353 – 288.1) = 64.9

E-factor = 64.9 /288.1 = 0.22 $KgKg^{-1}$

% Atom economy = 100 × $\frac{Molecular\ weight\ of\ the\ desired\ product}{Molecular\ weight\ all\ of\ reactants}$

% Atom economy = 100 × $\frac{335}{147 + 66 + 140}$ = 95%

Reaction mass efficiency (RME) = $\frac{mass\ of\ product}{\Sigma\ mass\ of\ stoichiometric\ reactants} \times\ 100$

Reaction mass efficiency (RME) = $\frac{288.1}{353} \times\ 100$ = 82%

Process mass intensity (PMI) = $\frac{\Sigma\,(mass\ of\ stoichiometric\ reactants\ +\ solvent}{mass\ of\ product}$

PMI = $\frac{353+3}{288.1}$ = 1.23

Ideal value of PMI = E-factor + 1 = 0.22 + 1 =1.22

E-score has been calculated for the reaction based on the following 6 parameters below (See Beilstein Journal of Organic Chemistry 2006, 2, 3.)

Entry	Parameter	Values	Penalty Points
1	Yield	(100-86)/2	7
2	Cost of reactants	Inexpensive	0
3	Safety of reactants	0+0+5	5
4	Technical setup	Common setup	0
5	Temperature /time	60 °C, >1 h	3
6	Workup and purification	Classical chromatography	1
Total penalty points			16

Eco-Score = 100 - the sum of individual penalties = 100-16 = 84

Eco-scale from 0 to 100 using the following scores: > 75, excellent; > 50, acceptable; and < 50, inadequate.

Characterization of the products

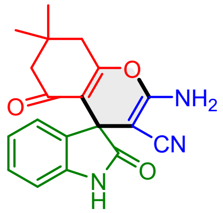

2-amino-7,7-dimethyl-2',5-dioxo-5,6,7,8-tetrahydrospiro[chromene-4,3'-indoline]-3-carbonitrile *(**4a**)*

White powder, mp (288-291 ºC)[10]; FT-IR (KBr cm^{-1}): 3374, 3311, 3144, 2962, 2192, 1722,1657, 1608, 1469, 1219, 1053; ^{1}H NMR (DMSO-d_6, 400 MHz) δ (ppm) : 10.47 (s, 1H), 7.30 (s, 2H), 7.20 (t, *J* = 7.7 Hz, 1H), 7.05 (d, *J* = 7.3 Hz, 1H), 6.97 (d, *J* = 7.4 Hz, 1H), 6.86 (d, *J* = 7.8 Hz, 1H), 2.56–2.59 (m, *2H*), 2.29 – 2.10 (m, 2H), 1.08 (d, *J* = 9.7 Hz, 6H). ^{13}C NMR (101 MHz, DMSO) δ: 195.4, 178.6, 164.7, 159.3, 142.6, 134.9, 128.7, 123.5, 122.29, 117.9, 111.3, 109.8, 58.0, 50.5, 47.35, 32.5, 28.1, 27.5.

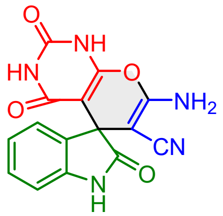

*7'-amino-2,2',4'-trioxo-1',2',3',4'-tetrahydrospiro[indoline-3,5'-pyrano[2,3-d]pyrimidine]-6'-carbonitrile (**4b**)*

White powder, mp (295-297 ºC)[11]; FT-IR (KBr cm^{-1}): 3352,3303, 3142, 2831, 2203, 1722,1674 ,1465, 1334, 1241, 1109^{1}H NMR (DMSO-d_6, 400 MHz) δ (ppm) : 11.13 (s, 1H), 10.49 (s, 1H), 7.37 (s, 2H), 7.15-7.35 (m, , 2H), 6.92 (t, *J* = 7.5 Hz, 1H), 6.80 (d, *J* = 7.7 Hz, 1H). ^{13}C NMR (101 MHz, DMSO) δ: 178.1, 161.9, 158.7, 153.8, 149.7, 142.5, 133.1, 128.9, 124.2, 122.3, 117.4, 109.8, 87.2, 58.2, 47.1.

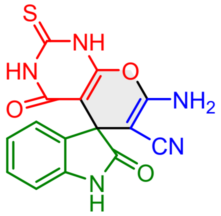

7'-amino-2,4'-dioxo-2'-thioxo-1',2',3',4'-tetrahydrospiro[indoline-3,5'-pyrano[2,3-d]pyrimidine]-6'-carbonitril ***(4c)***

White powder, mp (234-236 ºC)[12]; FT-IR (KBr cm^{-1}): 3435,3312, 3209, 2940 ,2197 ,175,1691, 1395, 1466,1239, 1046 ; ^1H NMR (DMSO-d_6, 400 MHz) : δ (ppm) δ 12.50 (s, 1H), 10.55 (s, 1H), 7.43 (s, 2H), 7.18 (t, J = 7.8 Hz, 2H), 6.92 (t, J = 7.5 Hz, 1H), 6.80 (d, J = 7.6 Hz, 1H); ^{13}C NMR (101 MHz, DMSO) δ 177.7, 174.4, 159.6, 158.6, 153.2, 142.6, 133.4, 129.1, 124.5, 122.3, 117.3, 109.8, 92.0, 57.9, 47.1.

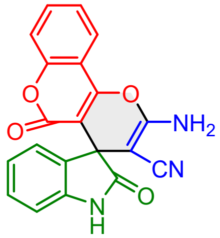

2'-amino-2,5'-dioxo-5'H-spiro[indoline-3,4'-pyrano[3,2-c]chromene]-3'-carbonitrile ***(4d)***

White powder, mp (288-290 ºC)[13]; FT-IR (KBr cm^{-1}): 3371, 3302, 3201, 3050 ,2202, 1718,1670, 1606, 1470, 1357, 1218, 1082 ; ^1H NMR (DMSO-d_6, 400 MHz) δ (ppm) : 10.72 (s, 1H), 7.97 (d, J = 7.9 Hz, 1H), 7.80 (s, 1H), 7.71 (s, 2H), 7.58 (d, J = 7.6 Hz, 1H), 7.52 (d, J = 8.3 Hz, 1H), 7.24 (d, J = 6.8 Hz, 2H), 7.02 – 6.85 (m, 2H). ^{13}C NMR (101 MHz, DMSO) δ: 177.6, 158.1, 155.6, 152.5, 142.7, 134.2, 133.5, 129.4, 125.5, 124.6, 123.2, 122.55, 117.5, 112.1, 110.0, 101.9, 57.5, 48.1.

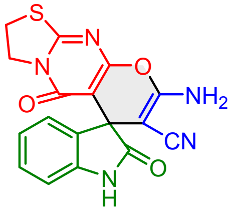

*8'-amino-2,5'-dioxo-2',3'-dihydro-5'H-spiro[indoline-3,6'-pyrano[2,3-d]thiazolo[3,2-a]pyrimidine]-7'-carbonitrile (**4e**)*

White powder, mp (290-293 ºC)[14]; FT-IR (KBr cm^{-1}): 3335, 3167, 3202, 3050 ,2182, 1716,1670, 1606, 1470, 1357, 1208, 1088 ;^{1}H NMR (DMSO- d_6, 400 MHz) : 10.52 (s, 1H), 7.36 (s, 2H), 7.16 (t, 1H, J = 7.6 Hz), 7.05 (d, 1H, J = 7.3 Hz), 6.90 (t, 1H, J = 7.5 Hz), 6.80 (d, 1H ,J = 7.7 Hz), 4.20 (t, 2H J = 7.7 Hz), 3.57 – 3.49 (m, 2H). ^{13}C NMR (101 MHz, DMSO) δ: 177.1, 166.2, 160.5, 159.1, 158.5, 142.7, 133.1, 128.1, 124.3, 122.3, 117.8, 109.7, 95.8, 57.0, 49.2, 27.3.

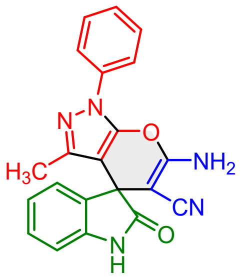

*6'-amino-3'-methyl-2-oxo-1'-phenyl-1'H-spiro[indoline-3,4'-pyrano[2,3-c]pyrazole]-5'-carbonitrile (**4f**)*

White powder, mp (225-227 ºC)[15]; FT-IR (KBr cm^{-1}): 3451, 3254, 3080, 2247, 1891, 1739, 1562, 1428, 1348, 1236, 1099, 988, 931 ; ^{1}H NMR (DMSO-d_6, 400 MHz) δ (ppm) : 10.76 (s, 1H), 7.80 (d, J = 8.6 Hz, 2H), 7.59 (s, 2H), 7.55 (s, 2H), 7.36 (t, J = 7.5 Hz, 1H), 7.29 (t, J = 7.6 Hz, 1H), 7.19 (d, J = 7.3 Hz, 1H), 7.04 (t, J = 7.5 Hz, 1H), 6.95 (d, J = 7.8 Hz, 1H), 1.55 (s, 3H). ^{13}C NMR (101 MHz, DMSO) δ: 177.1, 161.5, 145.3, 144.4, 142.1, 137.7, 132.6, 129.1, 129.8, 127.0, 125.4, 123.1, 120.6, 118.4, 110.3, 96.8, 56.6, 48.2, 12.2.

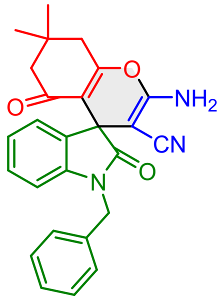

*2-Amino-1'-benzyl-7,7-dimethyl-2,5'-dioxo5',6',7',8'-tetrahydrospiro[chromene-4,3'-indoline]- 3-carbonitrile (**4g**)*

White solid, mp (265-267 ºC)[16]; FT-IR (KBr cm^{-1}): 3380, 3320, 3206, 2197, 1714, 1680, 1660, 1601, 1351, 1122 ;^{1}H NMR (DMSO-d_6, 400 MHz) δ (ppm) : 7.50 (d, J = 7.9 Hz, 2H), 7.35 (d, J = 6.1 Hz, 2H), 7.33 – 7.21 (m, 3H), 7.19 – 7.06 (m, 2H), 6.97 (t, J = 7.5 Hz, 1H), 6.69 (d, J = 7.7 Hz, 1H), 5.00 – 4.84 (m, 2H), 2.69 – 2.54 (m, 2H), 2.27 – 2.08 (m, 2H), 1.04 (d, J = 14.3 Hz, 6H). ^{13}C NMR (101 MHz, DMSO) δ: 195.5, 177.2, 165.0, 159.4, 143.1, 136.6, 134.1, 128.8, 128.7, 127.6, 127.6, 123.4, 123.0, 117.9, 111.1, 109.4, 57.6, 50.4, 47.0, 43.8, 32.5, 28.1, 27.5.

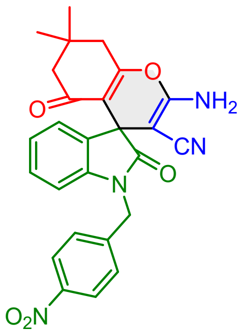

*2-amino-7,7-dimethyl-1'-(4-nitrobenzyl)-2',5-dioxo-5,6,7,8-tetrahydrospiro[chromene-4,3'-indoline]-3-carbonitrile (**4h**)*

Yellow solid, mp (302-304 ºC); FT-IR (KBr cm^{-1}): 3374, 3298, 3158, 2959 ,2878 ,2191 ,1725,1654 ,1603, 1483,1307,1243,1122,1045 ; ^{1}H NMR (DMSO-d_6, 400 MHz) δ (ppm) : 8.18 (d, J = 8.8 Hz, 2H), 7.78 (d, J = 8.8 Hz, 2H), 7.40 (s, 2H), 7.21 – 7.10 (m, 2H), 7.00 (t, J = 7.5 Hz, 1H), 6.78 (d, J = 7.8 Hz, 1H), 5.09 (s, 2H), 2.68 – 2.55 (m, 2H), 2.27 – 2.11 (m, 2H), 1.04 (d, J = 13.1 Hz, 6H). ^{13}C NMR (101 MHz, DMSO) δ: 195.8, 177.3, 165.2, 159.4, 147.3, 144.9, 142.6, 134.0, 128.9, 128.9 123.1, 123.6, 123.4, 117.1, 110.1, 109.2, 57.4, 50.3, 47.0, 43.3, 32.5, 28.1, 27.5.

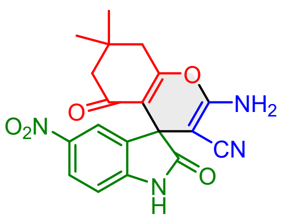

*2-amino-7,7-dimethyl-5'-nitro-2',5-dioxo-5,6,7,8-tetrahydrospiro[chromene-4,3'-indoline]-3-carbonitrile (**4i**)*

Yellow solid, mp (297-299ºC)[17]; FT-IR (KBr cm^{-1}): 3390, 3253, 3175, 2957, 2189, 1740,1643, 1590, 1467, 1520, 1342, 1221, 1059 ; ^{1}H NMR (DMSO-d_6, 400 MHz) δ (ppm) : 11.20 (s, 1H), 8.16 (dd, *J* = 8.6, 2.4 Hz, 1H), 7.97 (d, *J* = 2.3 Hz, 1H), 7.47 (s, 2H), 7.04 (d, *J* = 8.6 Hz, 1H), 2.53 (d, *J* = 12.5 Hz, 2H), 2.24 – 2.11 (m, 2H), 1.03 (s, 6H). ^{13}C NMR (101 MHz, DMSO) δ: 195.9, 179.1, 165.7, 159.6, 149.1, 142.9, 135.8, 126.3, 119.3, 117.5, 110.2, 109.1, 56.3, 50.3, 47.4, 32.5, 28.1, 27.5.

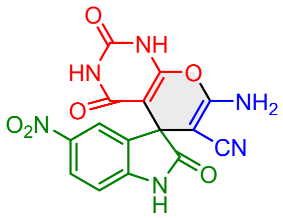

*7'-amino-5-nitro-2,2',4'-trioxo-1',2',3',4'-tetrahydrospiro[indoline-3,5'-pyrano[2,3-d]pyrimidine]-6'-carbonitrile (**4j**)*

Yellow solid, mp (230-232 ºC)[18]; FT-IR (KBr cm^{-1}): 3439,3301, 3205, 2939 ,2199 ,1749,1691, 1521, 1395, 1465, 1332, 1239, 1046 ; ^{1}H NMR (DMSO-d_6, 400 MHz) δ (ppm) : 11.24 (s, 1H), 11.20 (s, 1H), 8.25 (d, *J* = 2.3 Hz, 1H), 8.17 (dd, *J* = 8.6, 2.4 Hz, 1H), 7.57 (s, 2H), 7.02 (d, *J* = 8.6 Hz, 1H). ^{13}C NMR (101 MHz, DMSO) δ: 178.9, 162.1, 159.1, 154.4, 149.7, 149.1, 143.1, 135.1, 126.4, 120.5, 117.2, 109.1, 86.3, 56.6, 19.0.

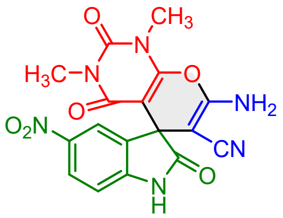

*7'-amino-1',3'-dimethyl-5-nitro-2,2',4'-trioxo-1',2',3',4'-tetrahydrospiro[indoline-3,5'-pyrano[2,3-d]pyrimidine]-6'-carbonitrile (**4k**)*

Yellow solid, mp (227-230 ºC)[19]; FT-IR (KBr cm^{-1}): 3482, 3351, 3174, 2196, 1692,1635, 1509, 1389, 1338 ,1193,1072 ; ^{1}H NMR (DMSO-d_6, 400 MHz) δ (ppm) : 11.28 (s, 1H), 8.18 (d, *J* = 11.6 Hz, 2H), 7.77 (s, 2H), 7.05 (d, *J* = 8.5 Hz, 1H), 3.40 (s, 3H), 3.03 (s, 3H). ^{13}C NMR (101 MHz, DMSO) δ: 178.7, 160.1, 158.9, 153.0, 150.1, 149.1, 143.0, 135.2, 126.5, 120.3, 117.1, 109.1, 86.5, 56.6, 28.1.

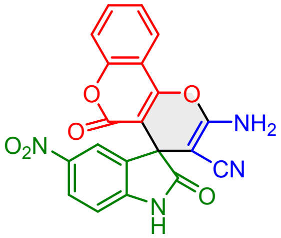

*2'-amino-5-nitro-2,5'-dioxo-5'H-spiro[indoline-3,4'-pyrano[3,2-c]chromene]-3'-carbonitrile (**4l**)*

White solid, mp (295-297 ºC)[20]; FT-IR (KBr cm^{-1}): 3380,3316, 3208, 2201, 1740,1672, 1610, 1469, 1521, 1354, 1214, 1099 ; ^{1}H NMR (DMSO-d_6, 400 MHz) δ (ppm) : 11.45 (s, 1H), 8.36 (d, *J* = 2.4 Hz, 1H), 8.22 (dd, *J* = 8.6, 2.4 Hz, 1H), 7.96 (dd, *J* = 8.0, 1.5 Hz, 1H), 7.86 (s, 2H), 7.80 – 7.73 (m, 1H), 7.59 – 7.45 (m, 2H), 7.10 (d, *J* = 8.6 Hz, 1H). ^{13}C NMR (101 MHz, DMSO) δ: 178.4, 159.3, 156.3, 152.6, 149.1, 143.2, 134.5, 126.8, 125.5, 123.3, 121.0, 117.2, 113.1, 110.2, 100.7, 56.5, 48.3.

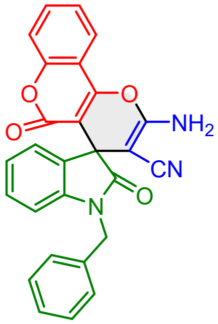

*2'-amino-1-benzyl-2,5'-dioxo-5'H-spiro[indoline-3,4'-pyrano[3,2-c]chromene]-3'-carbonitrile (**4m**)*

White solid solid, mp (281-283 ºC)[21]; FT-IR (KBr cm^{-1}): 3525, 3430, 3321, 3155, 2200, 1696, 1655, 1630, 1560, 1400, 1345, 11344 ; ^{1}H NMR (DMSO-d_6, 400 MHz) δ (ppm) : 7.98 (d, *J* = 7.9 Hz, 1H),

7.84 – 7.74 (m, 3H), 7.61 – 7.46 (m, 4H), 7.38 – 7.19 (m, 5H), 7.01 (t, *J* = 7.0 Hz, 1H), 6.82 (d, *J* = 7.9 Hz, 1H), 5.06 – 4.91 (m, 2H). ^{13}C NMR (101 MHz, DMSO) δ: 176.4, 159.1, 158.9, 155.8, 152.6, 143.2, 136.4, 134.3, 132.7, 129.5, 128.9, 127.7, 127.6, 125.6, 124.6, 123.4, 123.2, 117.5, 117.2, 112.1, 109.6, 101.6, 57.2, 47.8, 43.9.

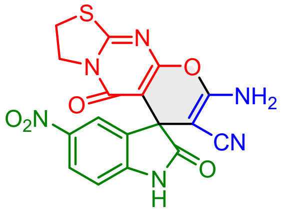

*8'-amino-5-nitro-2,5'-dioxo-2',3'-dihydro-5'H-spiro[indoline-3,6'-pyrano[2,3-d]thiazolo[3,2-a]pyrimidine]-7'-carbonitrile (**4n**)*

Yellow solid, mp (288-291 ºC); FT-IR (KBr cm^{-1}): 3331), 3203, 2202, 1718,1671, 1609, 1471, 1361, 1222, 1088 ; ^{1}H NMR (DMSO-d_6, 400 MHz) δ (ppm) : 11.30 (s, 1H), 8.18 (dd, *J* = 8.6, 2.4 Hz, 1H), 8.10 (d, *J* = 2.4 Hz, 1H), 7.56 (s, 2H), 7.04 (d, *J* = 8.6 Hz, 1H), 4.23 – 4.18 (m, 2H), 3.54 (d, *J* = 7.9 Hz, 2H). ^{13}C NMR (101 MHz, DMSO) δ: 178.7, 166.8, 160.8, 160.4, 158.7, 149.2, 143.0, 134.9, 126.5, 120.3, 117.6, 109.9, 94.7, 55.5, 49.3, 27.4. Anal.

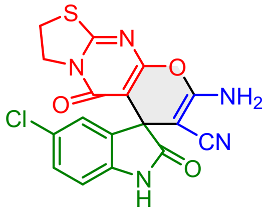

*8'-amino-5-chloro-2,5'-dioxo-2',3'-dihydro-5'H-spiro[indoline-3,6'-pyrano[2,3-d]thiazolo[3,2-a]pyrimidine]-7'-carbonitrile (**4o**)*

White powder mp (290-293 ºC)[22]; FT-IR (KBr cm^{-1}): 3358,3153, 3102, 2851, 2193, 1722,1674 ,1465, , 1252, 1089 ; ^{1}H NMR (DMSO-d_6, 400 MHz) δ (ppm) : 10.68 (s, 1H), 7.45 (s, 2H), 7.33 –

7.04 (m, 2H), 6.83 (d, *J* = 8.9 Hz, 1H), 4.23 (t, *J* = 8.1 Hz, 2H), 3.54 (t, *J* = 8.1 Hz, 2H). ^{13}C NMR (101 MHz, DMSO) δ: 177.8, 166.5, 160.7, 160.1, 158.6, 141.7, 135.9, 128.9, 126.3, 124.6, 117.8, 111.1, 95.2, 56.5, 49.3, 48.5, 27.4, 19.0.

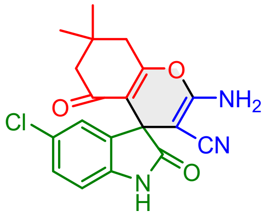

*2-Amino-5'-chloro-2',5-dioxo-7,7-dimethyl-5,6,7,8-tetrahydrospiro[chromene-4,3'-indoline]-3-carbonitrile. (**4p**)*

White powder, mp (291-293 ºC)[23]; FT-IR (KBr cm^{-1}): 3375, 3292, 2959, 2929, 2193, 1681, 1654, 1059 ; ^{1}H NMR (DMSO-d_6, 400 MHz) δ (ppm) : 10.55 (s, 1H), 7.32 (s, 2H), 7.19 (d, *J* = 8.3, Hz, 1H), 7.10 (S,1H), 6.81 (d, *J* = 8.2 Hz, 1H), 2.64 – 2.51 (m, 2H), 2.15 (d, *J* = 2.5 Hz, 2H), 1.02 (s, 6H). ^{13}C NMR (101 MHz, DMSO) δ: 195.6, 178.3, 165.1, 159.4, 141.5, 136.9, 128.6, 126.1, 123.8, 117.70, 111.1, 110.6, 57.2, 50.4, 47.6, 32.4, 27.1.

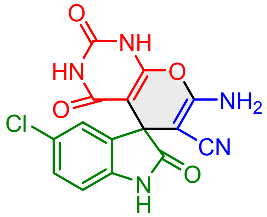

*7'-amino-5-chloro-2,2',4'-trioxo-1',2',3',4'-tetrahydrospiro[indoline-3,5'-pyrano[2,3-d]pyrimidine]-6'-carbonitrile. (**4q**)*

White powder, mp (230-232 ºC)[24]; FT-IR (KBr cm^{-1}): 32955, 3163, 3202, 3050 ,2182, 1716,1670, 1610, 1473, 1367, 1211, 999 ; ^{1}H NMR (DMSO-d_6, 400 MHz) δ (ppm) : 12.29 (s, 1H), 11.16 (s, 1H), 10.63 (s, 1H), 7.45 (s, 2H), 7.34 (s, 1H), 7.22 (s, 1H), 6.83 (d, *J* = 27.6 Hz, 1H). ^{13}C NMR (101 MHz, DMSO) δ :177.1, 162.0, 158.9, 154.0, 149.7, 141.5, 136.1, 128.8, 126.3, 124.6, 117.3, 111.1, 86.7, 57.5.

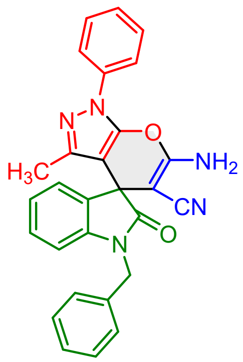

*6'-amino-3'-methyl-2-oxo-1'-phenyl-1'H-spiro[1-benzylindoline-3,4'-pyrano[2,3-c]pyrazole]-5'-carbonitrile (**4r**)*

Yellow solid, mp (230-232 ºC)[25]; FT-IR (KBr cm^{-1}): 3369,3296, 3202, 2202, 1719,1670, 1606, 1480, 1360, 1227, 1108 ; ^{1}H NMR (DMSO-d_6, 400 MHz) δ (ppm) : 7.80 (d, J = 8.8 Hz, 2H), 7.68 (s, 2H), 7.53 (t, J = 8.0 Hz, 2H), 7.44 (d, J = 7.8 Hz, 2H), 7.40 – 7.26 (m, 6H), 7.10 (t, J = 7.0 Hz, 2H), 5.10 – 4.93 (m, 2H), 1.36 (s, 3H). ^{13}C NMR (101 MHz, DMSO) δ: 176.6, 161.6, 145.5, 144.3, 142.6, 137.7, 136.5, 131.8, 129.9, 129.9, 129.1, 128.0, 127.1, 125.3, 123.9, 120.7, 118.4, 109.1, 96.6, 56.3, 47.1, 43.8, 12.2.

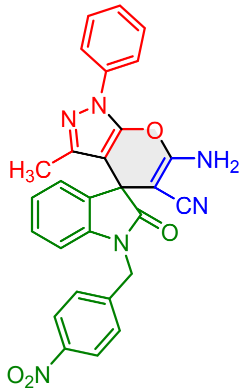

*6'-amino-3'-methyl-1-(4-nitrobenzyl)-2-oxo-1'-phenyl-1'H-spiro[indoline-3,4'-pyrano[2,3-c]pyrazole]-5'-carbonitrile (**4s**)*

Yellow solid, mp (268-270ºC); FT-IR (KBr cm^{-1}): 3331, 3203, 2202, 1718,1671 ,1609, 1471,1404, 1361, 1222, 1088 ,950 ; ^{1}H NMR (DMSO-d_6, 400 MHz) δ (ppm) : 8.22 (d, J = 8.7 Hz, 2H), 7.81 (d, J = 8.0 Hz, 2H), 7.71 (d, J = 8.9 Hz, 4H), 7.53 (t, J = 7.9 Hz, 2H), 7.42 – 7.24 (m, 3H), 7.19 – 7.06 (m, 2H), 5.28 – 5.08 (m, 2H), 1.42 (s, 3H). ^{13}C NMR (101 MHz, DMSO) δ: 176.8, 161.6, 147.5,

145.5, 144.4, 144.3, 142.2, 137.6, 131.8, 129.1, 129.3, 127.2, 125.5, 124.2, 120.7, 118.6 109.9, 96.3, 56.2, 47.1, 43.2, 12.3.

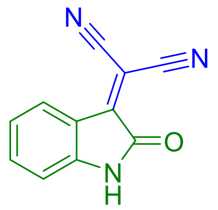

*2-(2-oxo-indolin-3-ylidene)malononitrile(**II**)*

Orchid purple, mp (235-237ºC)[26], FT-IR (KBr cm^{-1}): 3245, 3107, 2231, 1714, 1614, 1578, 1342, ^{1}H NMR (DMSO-d_6, 400 MHz)δ 11.22 (s, 1H), 7.88 (d, J = 7.8 Hz, 1H), 7.58 (t, J = 7.6 Hz, 1H), 7.14 (t, J = 7.7 Hz, 1H), 6.94 (d, J = 7.9 Hz, 1H). ^{13}C NMR (101 MHz, DMSO) δ 164.22, 151.09, 146.94, 138.29, 126.29, 123.39, 119.11, 113.53, 112.11, 81.07

.

References

(1) Bruckman, V. J.; Wriessnig, K. Improved Soil Carbonate Determination by FT-IR and X-Ray Analysis. *Environ. Chem. Lett.* **2013**, *11* (1), 65–70. https://doi.org/10.1007/s10311-012-0380-4.

(2) Boeriu, C. G.; Bravo, D.; Gosselink, R. J. A.; Van Dam, J. E. G. Characterisation of Structure-Dependent Functional Properties of Lignin with Infrared Spectroscopy. *Ind. Crops Prod.* **2004**, *20* (2), 205–218. https://doi.org/10.1016/j.indcrop.2004.04.022.

(3) Cao, X.; Harris, W. Properties of Dairy-Manure-Derived Biochar Pertinent to Its Potential Use in Remediation. *Bioresour. Technol.* **2010**, *101* (14), 5222–5228. https://doi.org/10.1016/j.biortech.2010.02.052.

(4) Han, L.; Qian, L.; Liu, R.; Chen, M.; Yan, J.; Hu, Q. Lead Adsorption by Biochar under the Elevated Competition of Cadmium and Aluminum. *Sci. Rep.* **2017**, *7* (1), 2264. https://doi.org/10.1038/s41598-017-02353-4.

(5) Zhang, S.-Z.; Cui, Z.-S.; Zhang, M.; Zhang, Z.-H. Biochar-Based Functional Materials as Heterogeneous Catalysts for Organic Reactions. *Curr. Opin. Green Sustain. Chem.* **2022**, *38*, 100713. https://doi.org/10.1016/j.cogsc.2022.100713.

(6) Xiang, Y.; Zheng, Y.; Yang, L.; Li, M.; Mao, Y.; Zhu, J. Exploring a New Dy3+-Activated Borotellurate Phosphor with Thermally Stable Photoluminescence. *J. Alloys Compd.* **2022**, *919*, 165837. https://doi.org/10.1016/j.jallcom.2022.165837.

(7) *Biochar for Environmental Management*, 0 ed.; Lehmann, J., Joseph, S., Eds.; Routledge, 2012. https://doi.org/10.4324/9781849770552.

(8) Akamatsu, N.; Nakajima, H.; Ono, M.; Miura, Y. Increase in Acetyl CoA Synthetase Activity after Phenobarbital Treatment. *Biochem. Pharmacol.* **1975**, *24* (18), 1725–1727. https://doi.org/10.1016/0006-2952(75)90013-1.

(9) Ro, K. S.; Cantrell, K. B.; Hunt, P. G. High-Temperature Pyrolysis of Blended Animal Manures for Producing Renewable Energy and Value-Added Biochar. *Ind. Eng. Chem. Res.* **2010**, *49* (20), 10125–10131. https://doi.org/10.1021/ie101155m.

(10) Bodhak, C.; Kundu, A.; Pramanik, A. ZrO_2 Nanoparticles as a Reusable Solid Dual Acid–Base Catalyst for Facile One-Pot Synthesis of Multi-Functionalized Spirooxindole Derivatives under Solvent Free Condition. *RSC Adv.* **2015**, *5* (104), 85202–85213. https://doi.org/10.1039/C5RA16259A.

(11) Jamatia, R.; Gupta, A.; Pal, A. K. Nano-FGT: A Green and Sustainable Catalyst for the Synthesis of Spirooxindoles in Aqueous Medium. *RSC Adv.* **2016**, *6* (25), 20994–21000. https://doi.org/10.1039/C5RA27552K.

(12) Jamatia, R.; Gupta, A.; Pal, A. K. Nano-FGT: A Green and Sustainable Catalyst for the Synthesis of Spirooxindoles in Aqueous Medium. *RSC Adv.* **2016**, *6* (25), 20994–21000. https://doi.org/10.1039/C5RA27552K.

(13) Thongni, A.; Phanrang, P. T.; Pandya, C.; Diengdoh, D. F.; Gannon, P. M.; Kaminsky, W.; Nongkhlaw, R.; Kumari, J.; Sriram, D.; Sivaramakrishna, A.; Nongkhlaw, R. Ultrasound Assisted Synthesis of Spirooxindole Analogs Catalyzed by Fe3O4@PPCA NPs: Experimental, Theoretical and in Vitro Biological Studies. *J. Mol. Struct.* **2023**, *1284*, 135363. https://doi.org/10.1016/j.molstruc.2023.135363.

(14) Nagaraju, S.; Paplal, B.; Sathish, K.; Giri, S.; Kashinath, D. Synthesis of Functionalized Chromene and Spirochromenes Using l -Proline-Melamine as Highly Efficient and Recyclable Homogeneous Catalyst at Room Temperature. *Tetrahedron Lett.* **2017**, *58* (44), 4200–4204. https://doi.org/10.1016/j.tetlet.2017.09.060.

(15) Esmaeili, A. A.; Amini-Ghalandarabad, S.; Mesbah, F.; Tasmimi, M.; Izadyar, M.; Fakhari, A. R.; Salimi, A. R. Efficient Synthesis of Novel Spiro[Indole-3,6′-Pyrano[2,3-d][1,3]Thiazolo[3,2-a]Pyrimidine Derivatives through an Organobase-Catalyzed, Three-Component Reaction. *Tetrahedron* **2015**, *71* (16), 2458–2462. https://doi.org/10.1016/j.tet.2015.01.055.

(16) Dalal, K. S.; Tayade, Y. A.; Wagh, Y. B.; Trivedi, D. R.; Dalal, D. S.; Chaudhari, B. L. Bovine Serum Albumin Catalyzed One-Pot, Three-Component Synthesis of Dihydropyrano[2,3-c]Pyrazole Derivatives in Aqueous Ethanol. *RSC Adv.* **2016**, *6* (18), 14868–14879. https://doi.org/10.1039/C5RA13014J.

(17) Raheja, B. K.; Dalal, D. S. Ammonium Acetate Mediated Simple, Rapid, and One-Pot Multicomponent Synthesis of Spirooxindole Derivatives. *Synth. Commun.* **2023**, *53* (11), 808–822. https://doi.org/10.1080/00397911.2023.2199357.

(18) Bodhak, C.; Kundu, A.; Pramanik, A. ZrO_2 Nanoparticles as a Reusable Solid Dual Acid–Base Catalyst for Facile One-Pot Synthesis of Multi-Functionalized Spirooxindole Derivatives under Solvent Free Condition. *RSC Adv.* **2015**, *5* (104), 85202–85213. https://doi.org/10.1039/C5RA16259A.

(19) Langroudi, J. M.; Mazloumi, M.; Nahzomi, H. T.; Shirini, F.; Tajik, H. Tropine-Based Dicationic Molten Salt: An Active Catalyst in the Acceleration of One-Pot Synthesis of Spiro-2-Amino-4H-Pyran-Oxindoles and Bis-2-Amino-4H-Pyrans. *J. Mol. Struct.* **2023**, *1274*, 134410. https://doi.org/10.1016/j.molstruc.2022.134410.

(20) Westphal, R.; Venturini Filho, E.; Loureiro, L. B.; Tormena, C. F.; Pessoa, C.; Guimarães, C. D. J.; Manso, M. P.; Fiorot, R. G.; Campos, V. R.; Resende, J. A. L. C.; Medici, F.;

Greco, S. J. Green Synthesis of Spiro Compounds with Potential Anticancer Activity through Knoevenagel/Michael/Cyclization Multicomponent Domino Reactions Organocatalyzed by Ionic Liquid and Microwave-Assisted. *Molecules* **2022**, *27* (22), 8051. https://doi.org/10.3390/molecules27228051.

(21) Guo, R.-Y.; An, Z.-M.; Mo, L.-P.; Wang, R.-Z.; Liu, H.-X.; Wang, S.-X.; Zhang, Z.-H. Meglumine: A Novel and Efficient Catalyst for One-Pot, Three-Component Combinatorial Synthesis of Functionalized 2-Amino-4 *H* -Pyrans. *ACS Comb. Sci.* **2013**, *15* (11), 557–563. https://doi.org/10.1021/co400107j.

(22) Langroudi, J. M.; Mazloumi, M.; Nahzomi, H. T.; Shirini, F.; Tajik, H. Tropine-Based Dicationic Molten Salt: An Active Catalyst in the Acceleration of One-Pot Synthesis of Spiro-2-Amino-4H-Pyran-Oxindoles and Bis-2-Amino-4H-Pyrans. *J. Mol. Struct.* **2023**, *1274*, 134410. https://doi.org/10.1016/j.molstruc.2022.134410.

(23) Wagh, Y. B.; Padvi, S. A.; Mahulikar, P. P.; Dalal, D. S. CsF Promoted Rapid Synthesis of Spirooxindole-pyran Annulated Heterocycles at Room Temperature in Ethanol. *J. Heterocycl. Chem.* **2020**, *57* (3), 1101–1110. https://doi.org/10.1002/jhet.3846.

(24) Saberi, D.; Bashkar, M.; Rezaei, A.; Azizi, Z.; Niknam, K. Guanine Base Stabilized on the Magnetic Nanoparticles as Recyclable Catalyst “on Water” for the Synthesis of Spirooxindole Derivatives. *J. Organomet. Chem.* **2021**, *948*, 121912. https://doi.org/10.1016/j.jorganchem.2021.121912.

(25) Dandia, A.; Saini, D.; Bhaskaran, S.; Saini, D. K. Ultrasound Promoted Green Synthesis of Spiro[Pyrano[2,3-c]Pyrazoles] as Antioxidant Agents. *Med. Chem. Res.* **2014**, *23* (2), 725–734. https://doi.org/10.1007/s00044-013-0671-8.

(26) Zhu, L.; Yan, P.; Zhang, L.; Chen, Z.; Zeng, X.; Zhong, G. $TiCl_4$ /DMAP Mediated Z-Selective Knovenagel Condensation of Isatins with Nitroacetates and Related Compounds. *RSC Adv.* **2017**, *7* (81), 51352–51358. https://doi.org/10.1039/C7RA09951G.

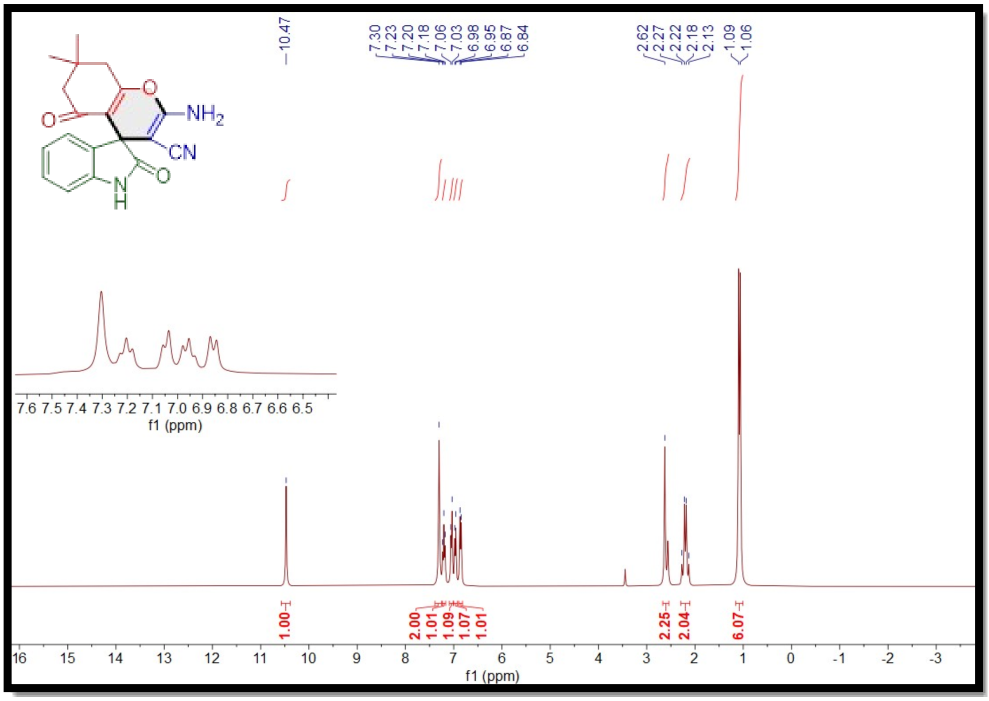

^{1}H-NMR of **4a**

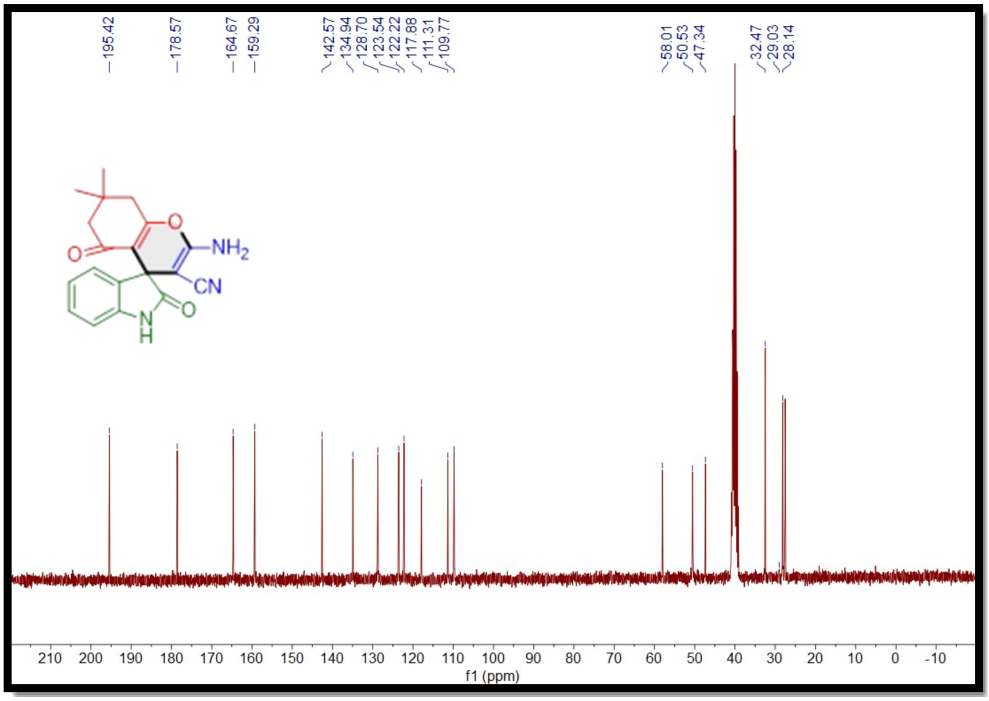

^{13}C-NMR of **4a**

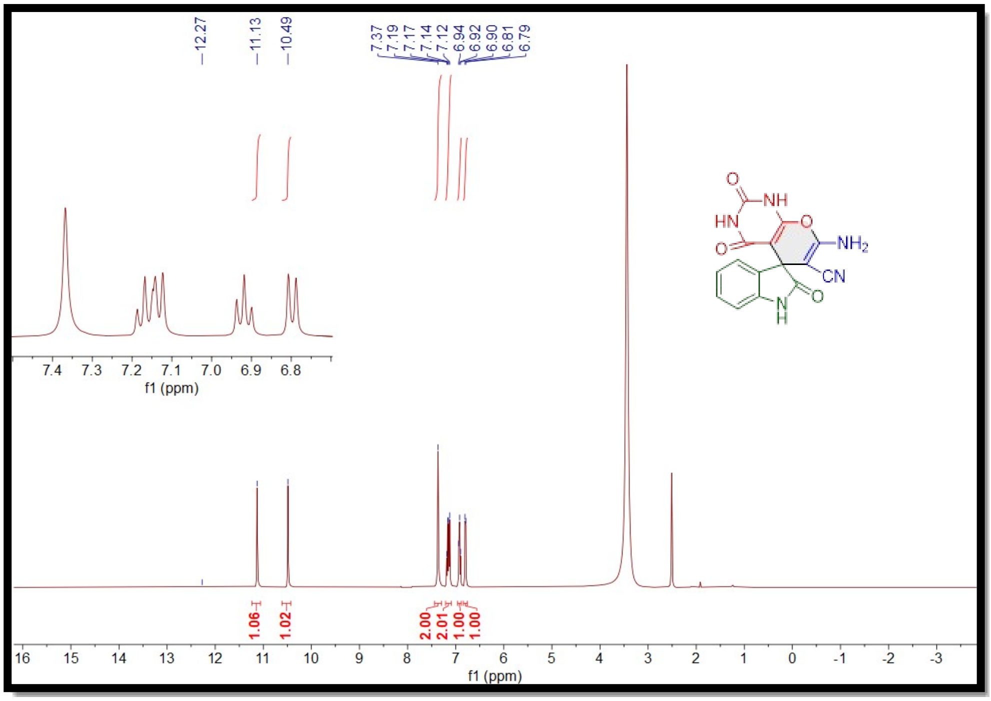

^{1}H-NMR of **4b**

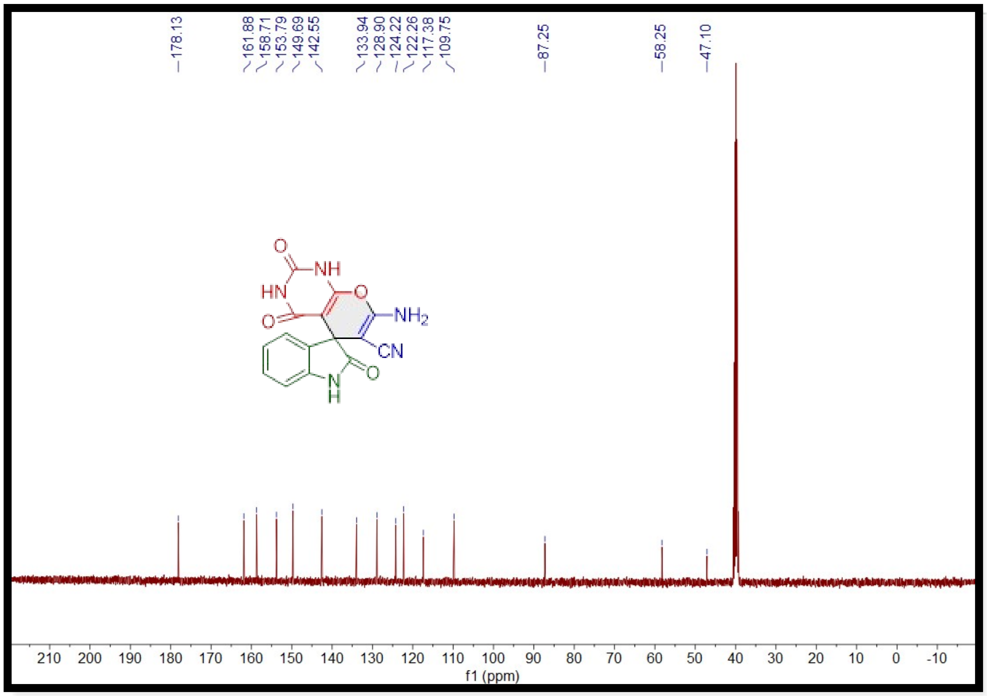

^{13}C-NMR of **4b**

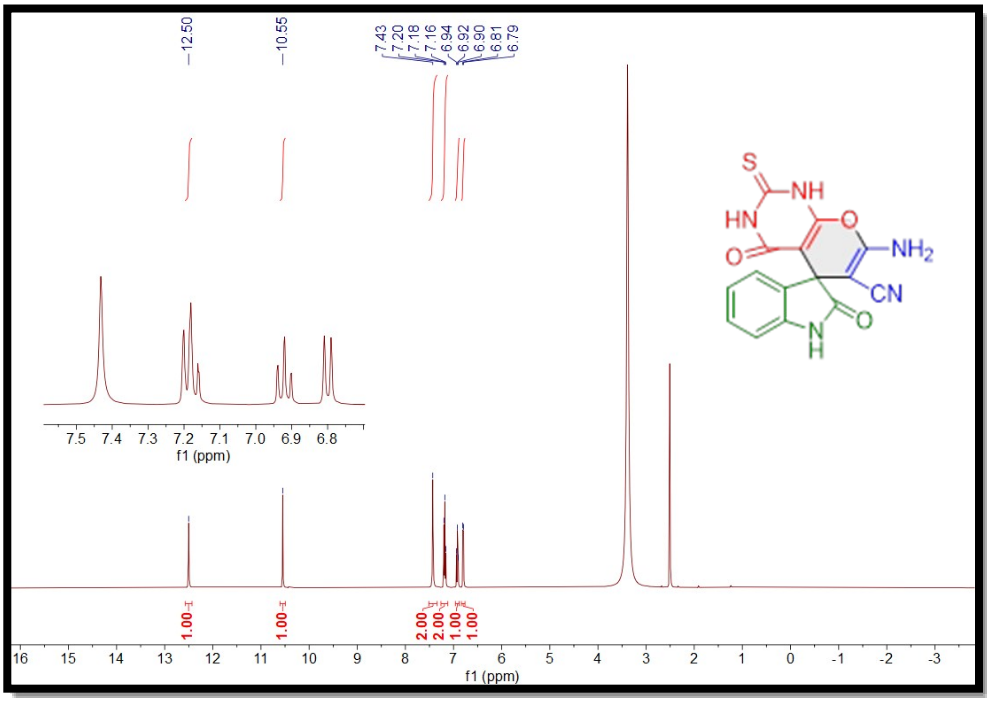

^{1}H-NMR of **4c**

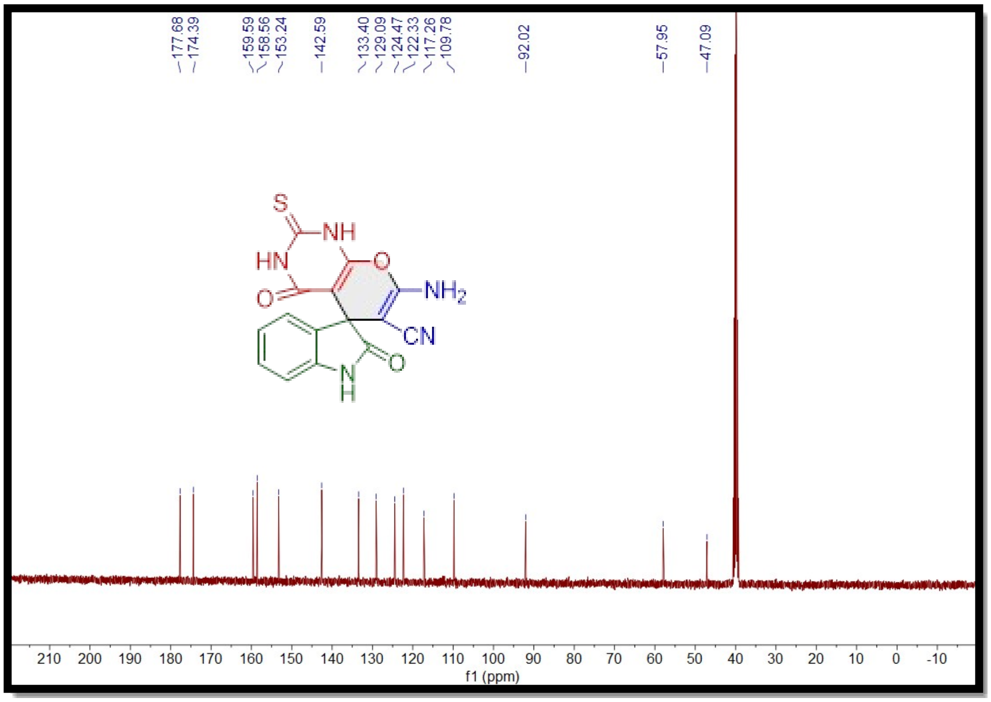

^{13}C-NMR of **4c**

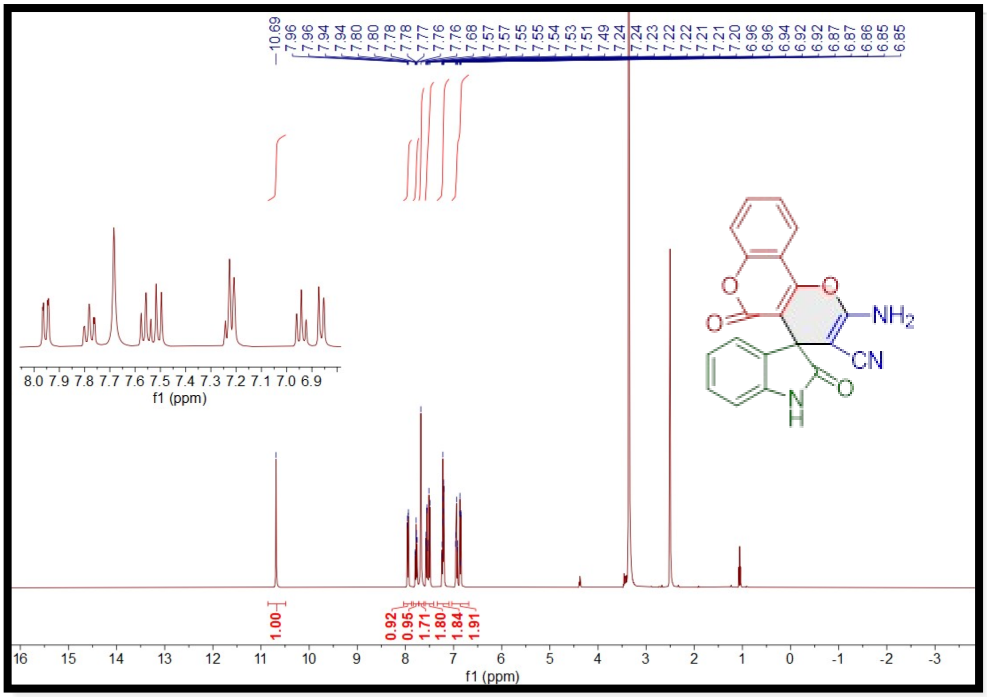

^{1}H-NMR of **4d**

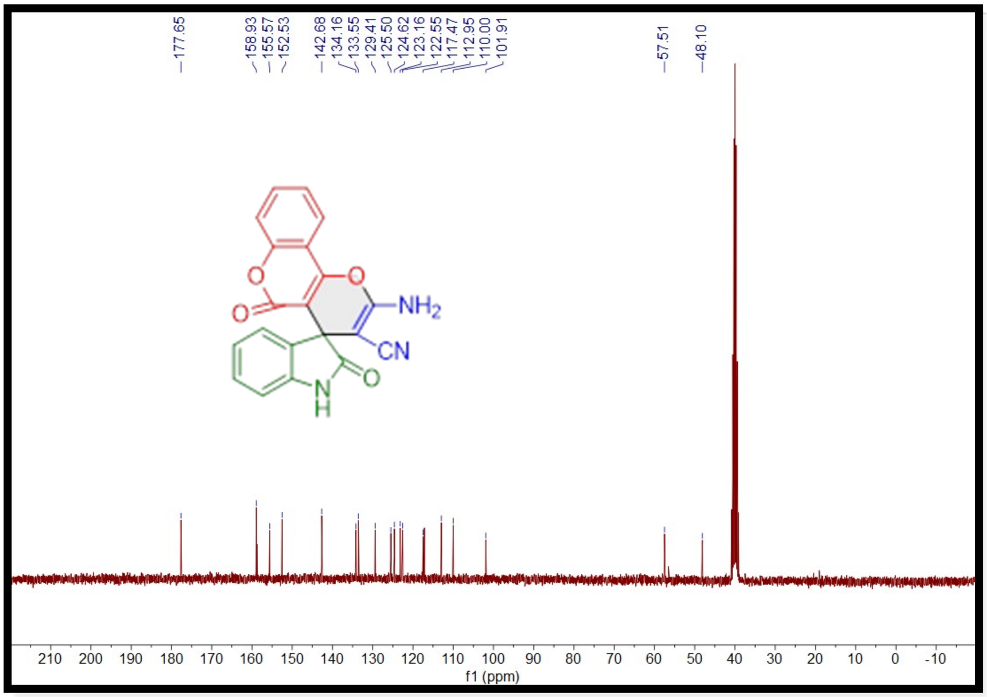

^{13}C-NMR of **4d**

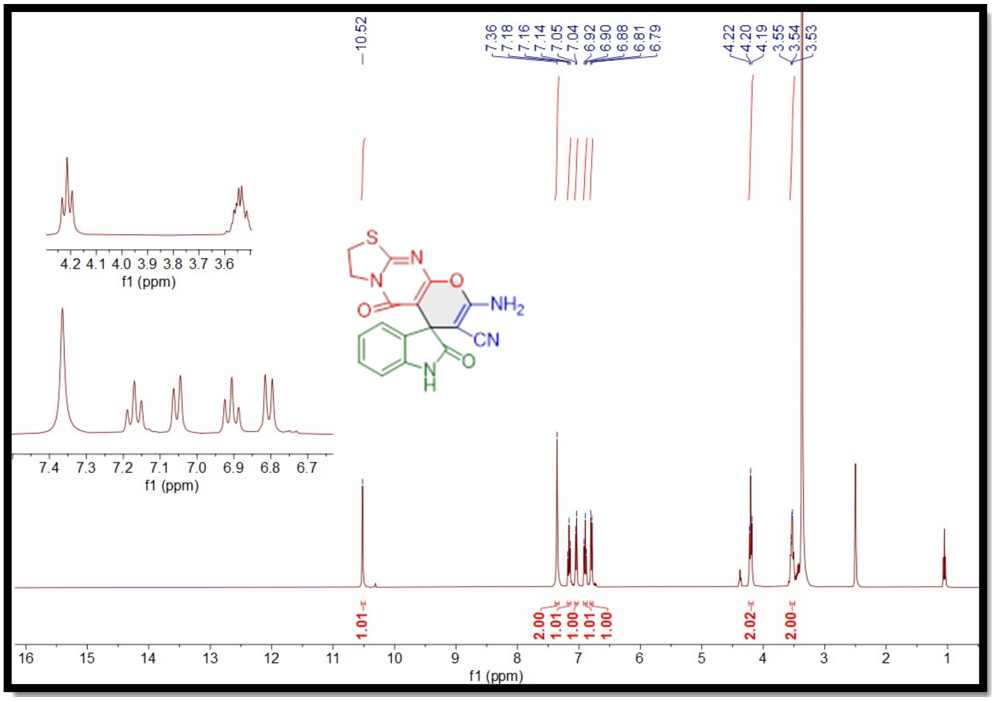

^{1}H-NMR of **4e**

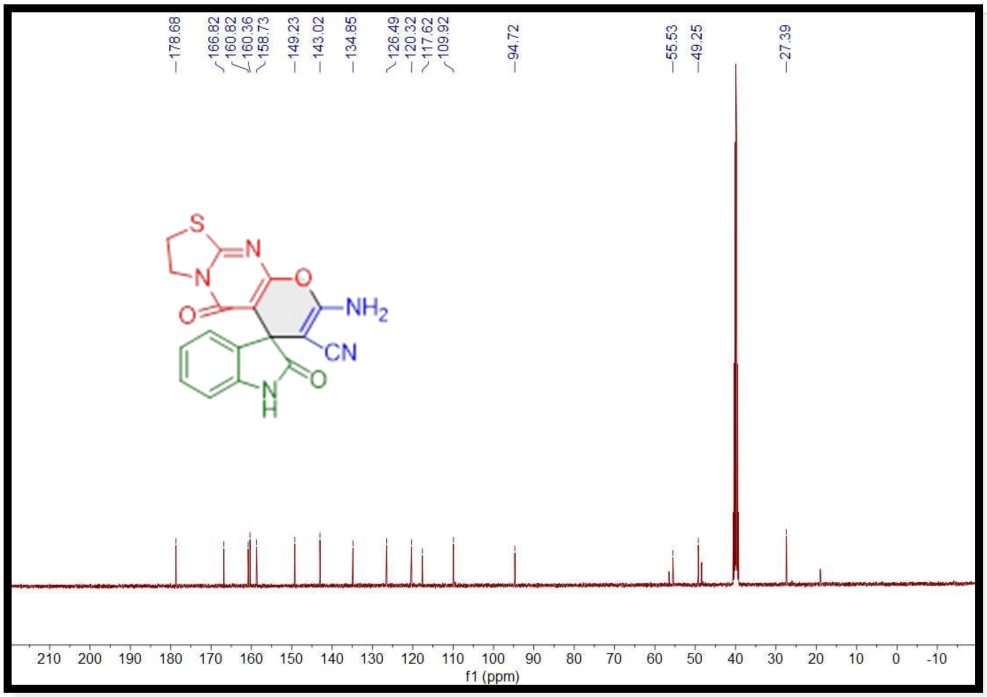

^{13}C-NMR of **4e**

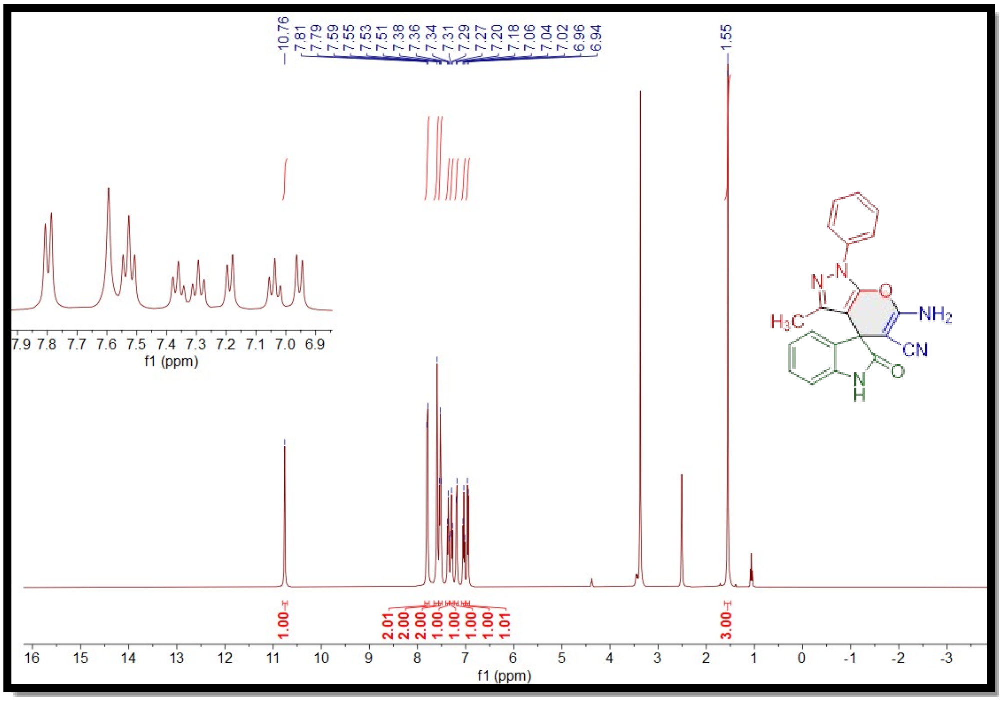

^{1}H-NMR of **4f**

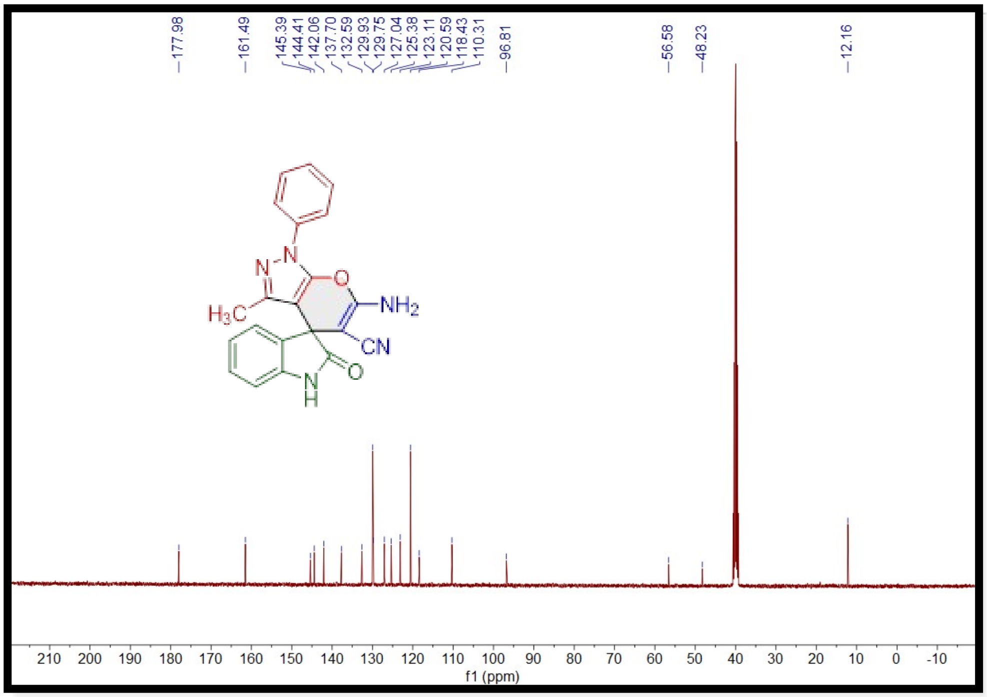

^{13}C-NMR of **4f**

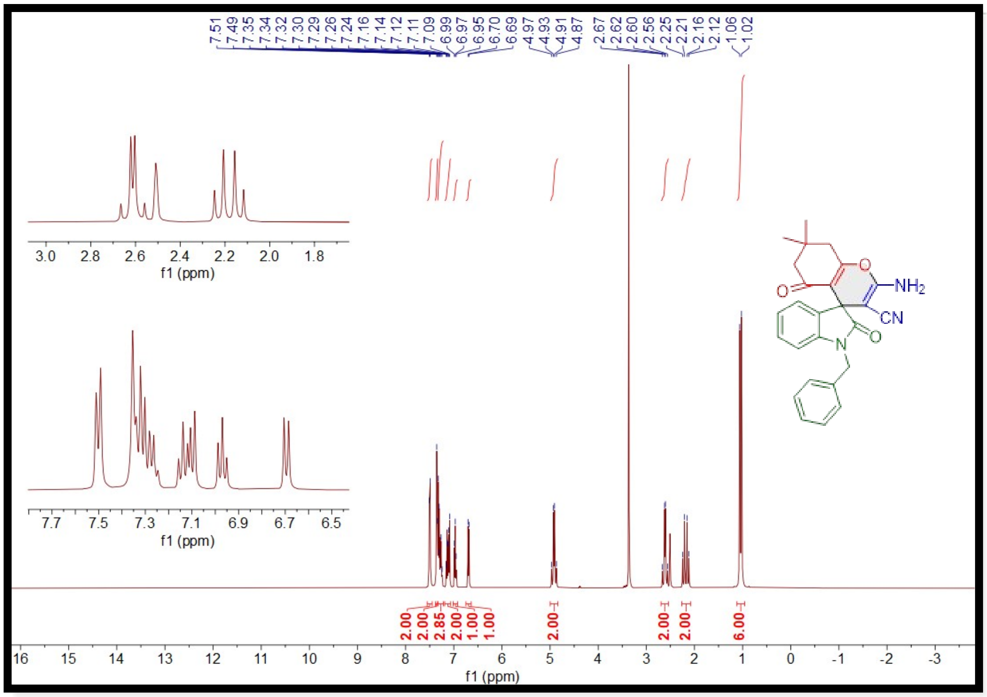

^{1}H-NMR of **4g**

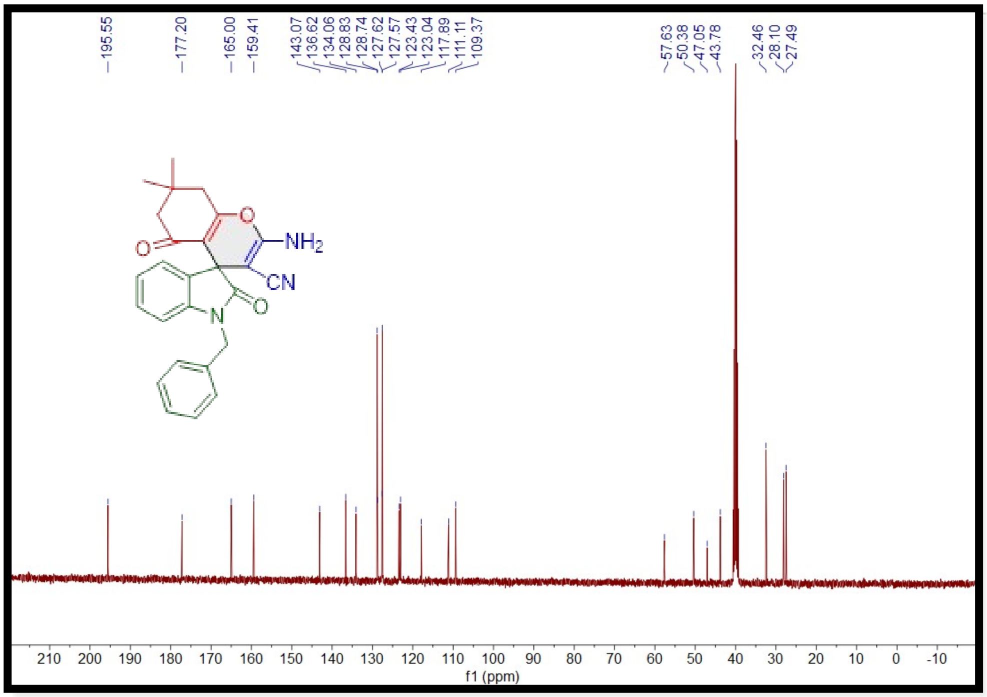

^{13}C-NMR of **4g**

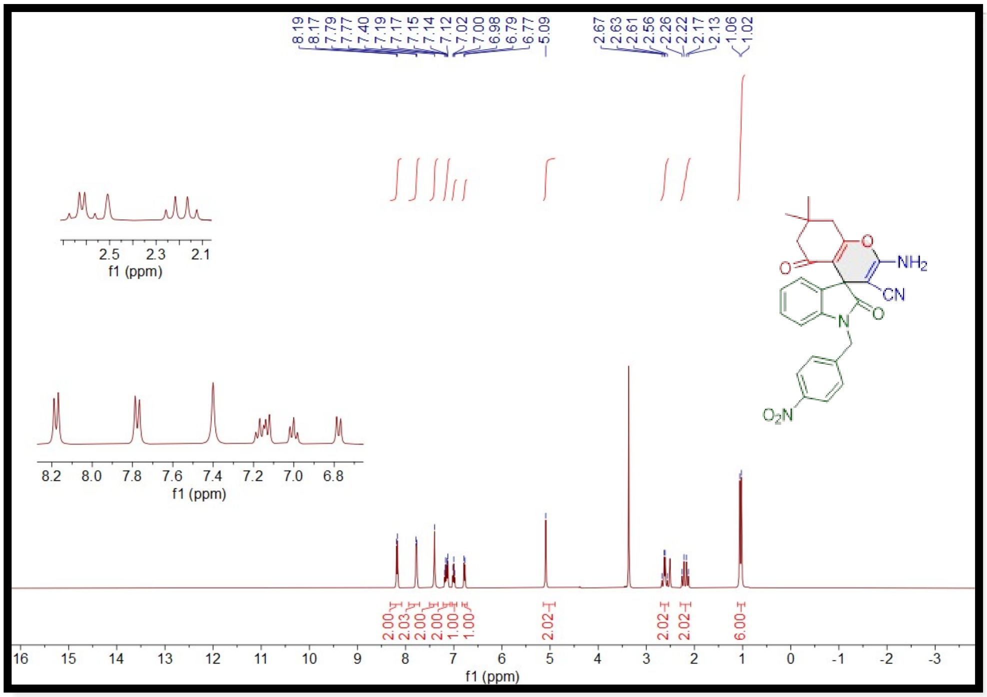

^{1}H-NMR of **4h**

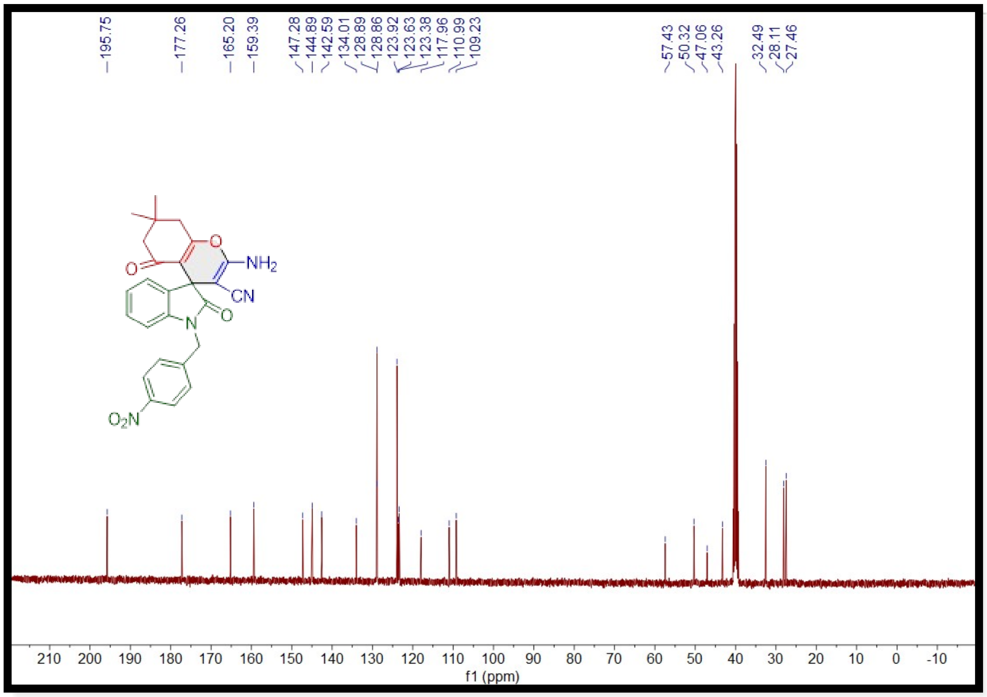

^{13}C-NMR of **4h**

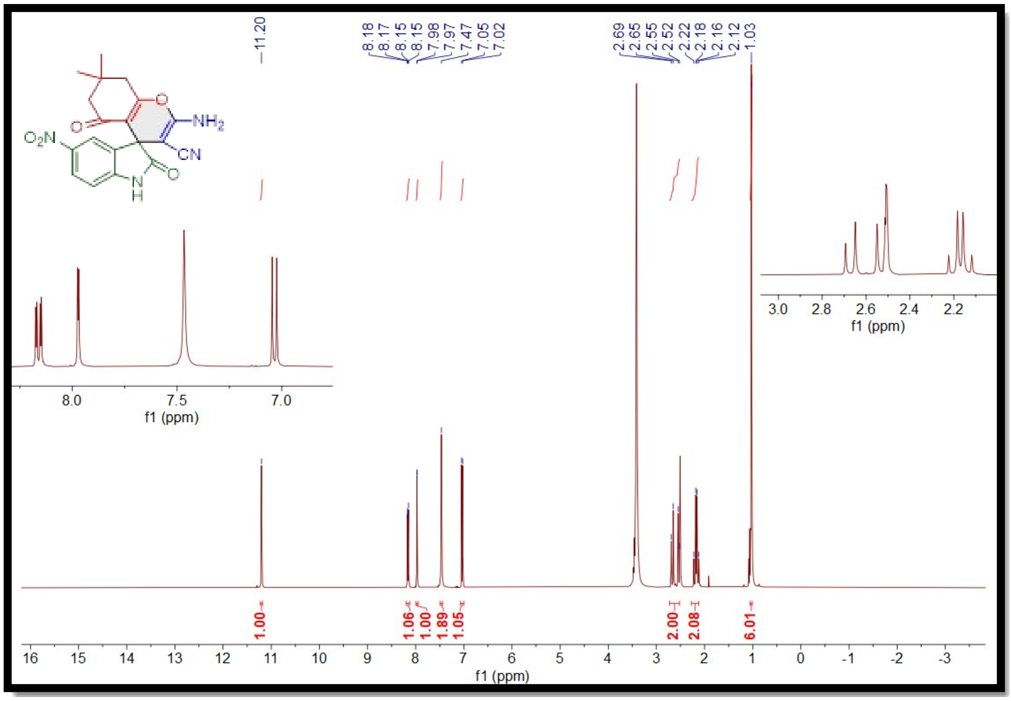

^{1}H-NMR of **4i**

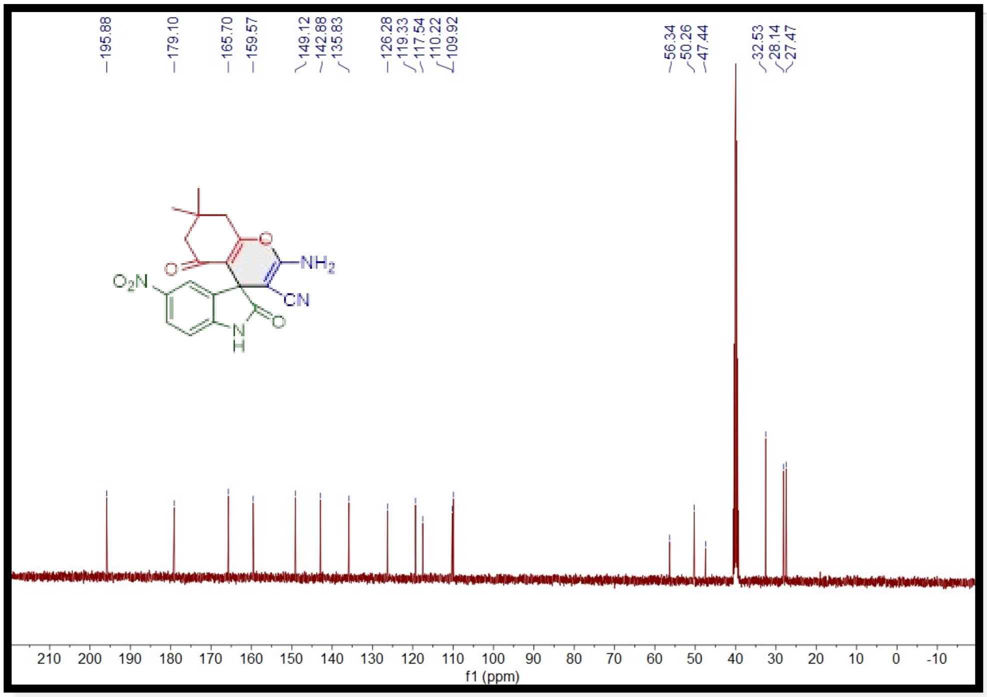

^{13}C-NMR of **4i**

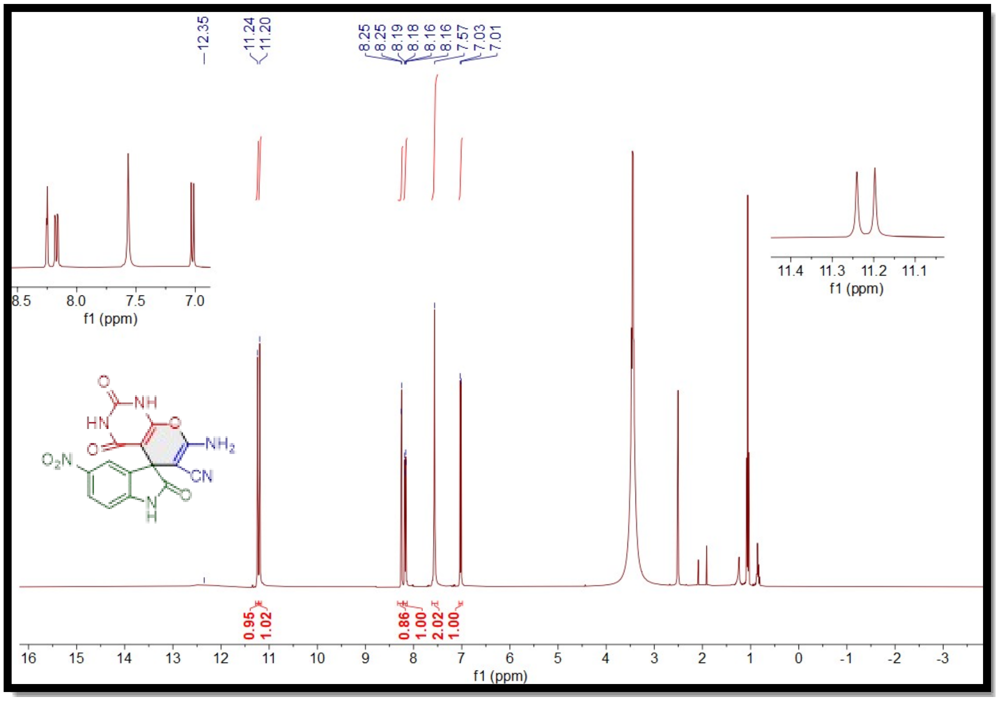

^{1}H-NMR of **4j**

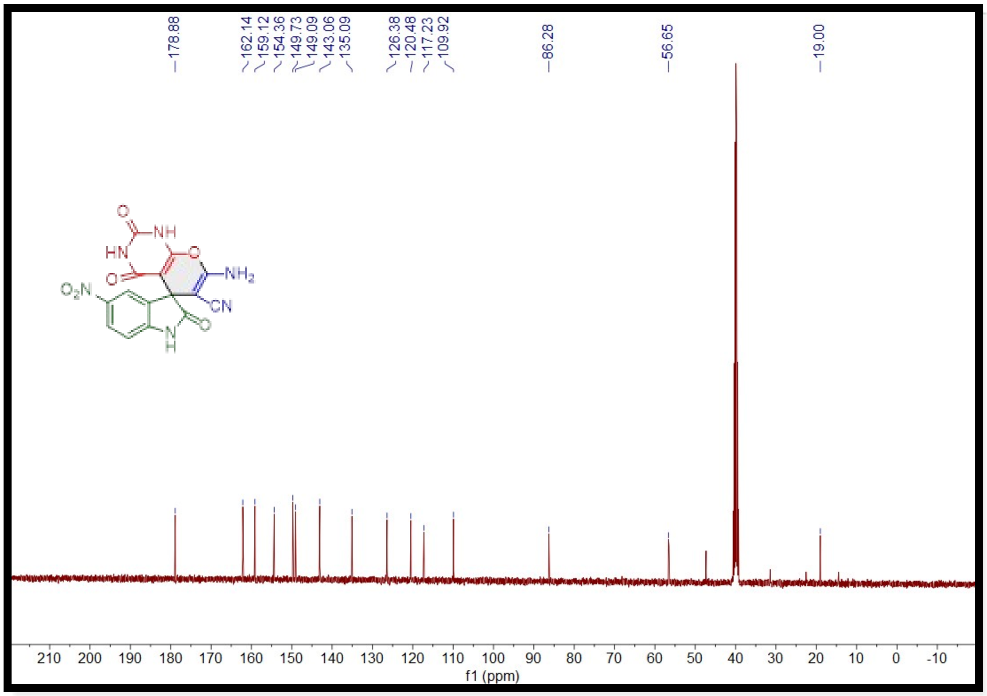

^{13}C-NMR of **4j**

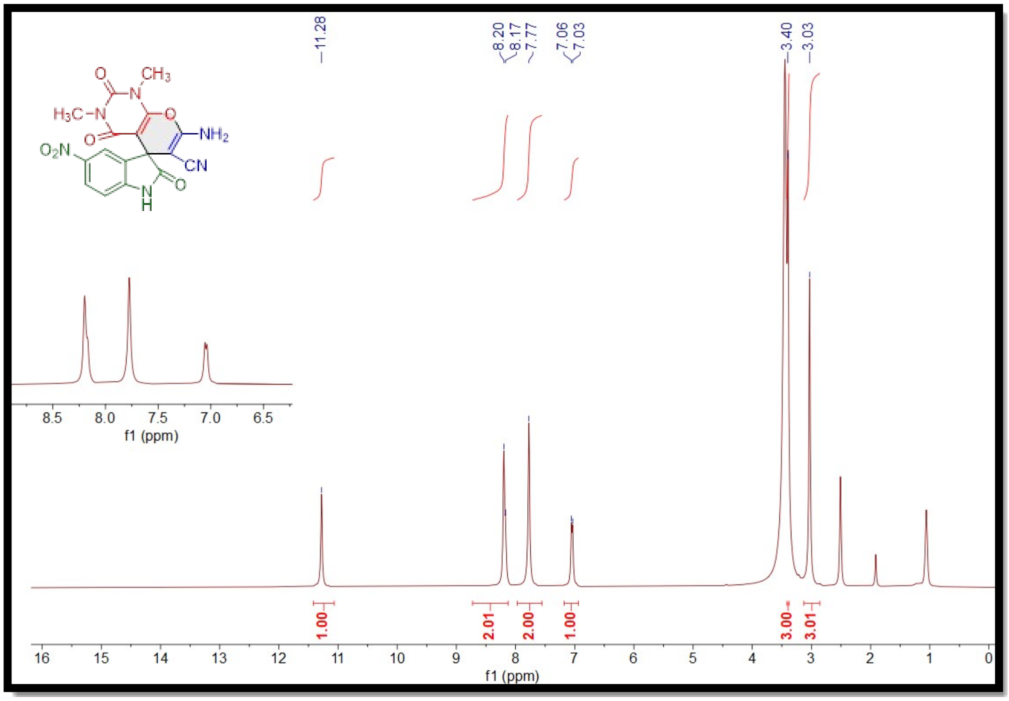

^{1}H-NMR of **4k**

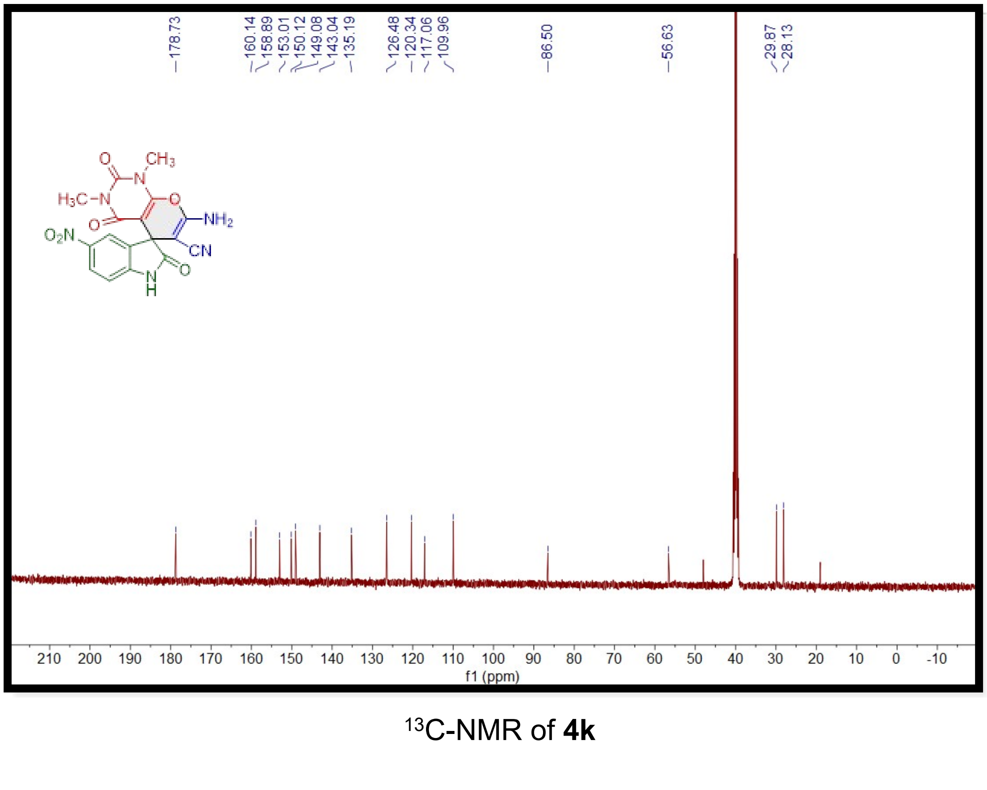

^{13}C-NMR of **4k**

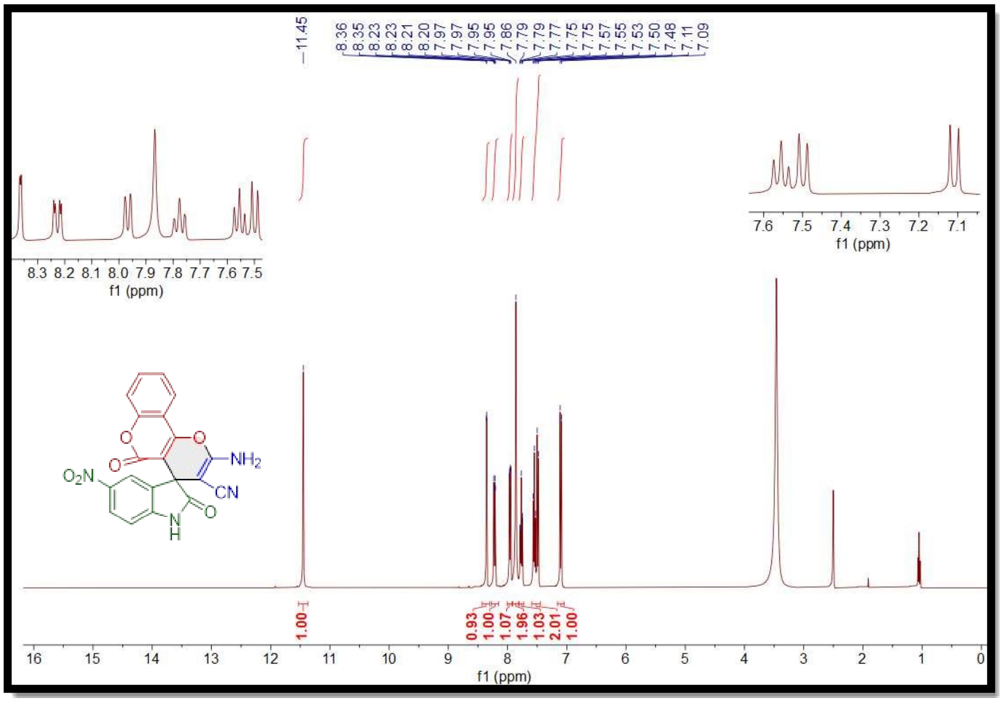

^{1}H-NMR of **4l**

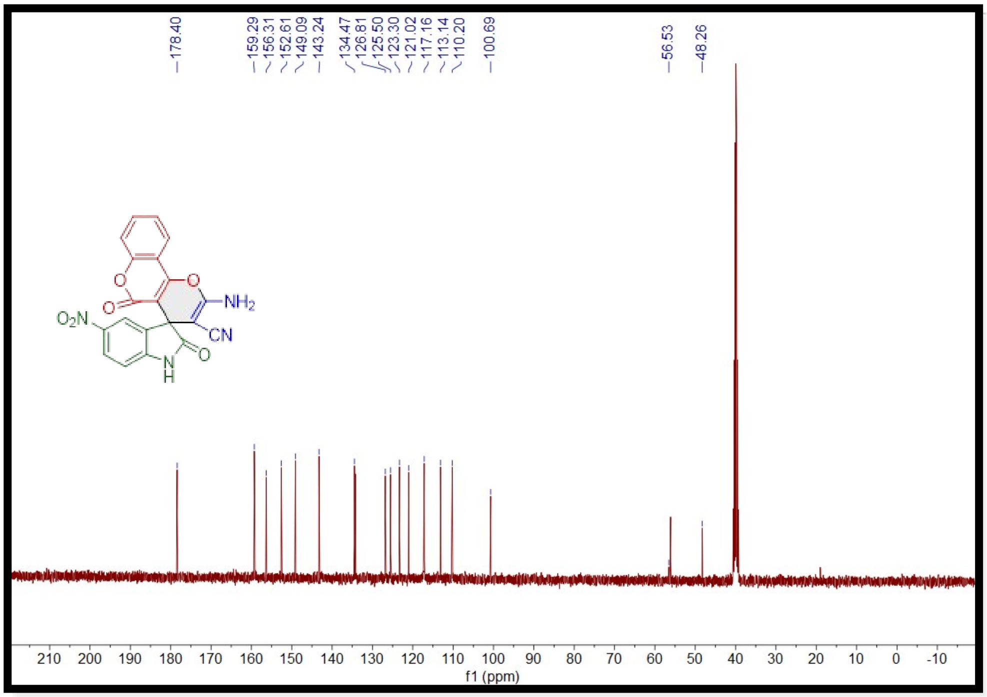

^{13}C-NMR of **4l**

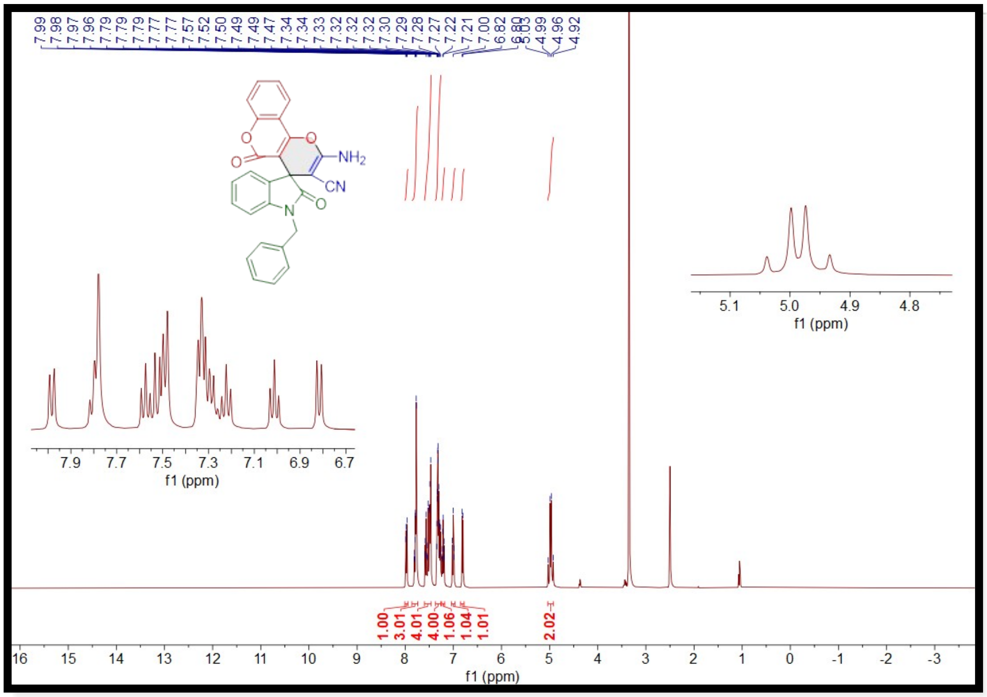

^{1}H-NMR of **4m**

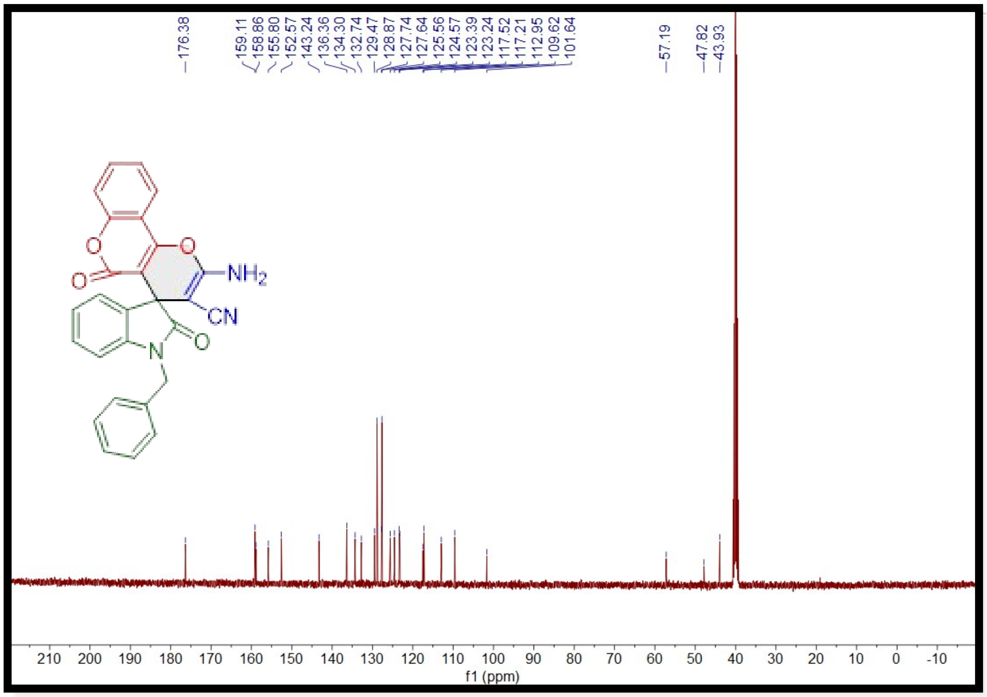

^{13}C-NMR of **4m**

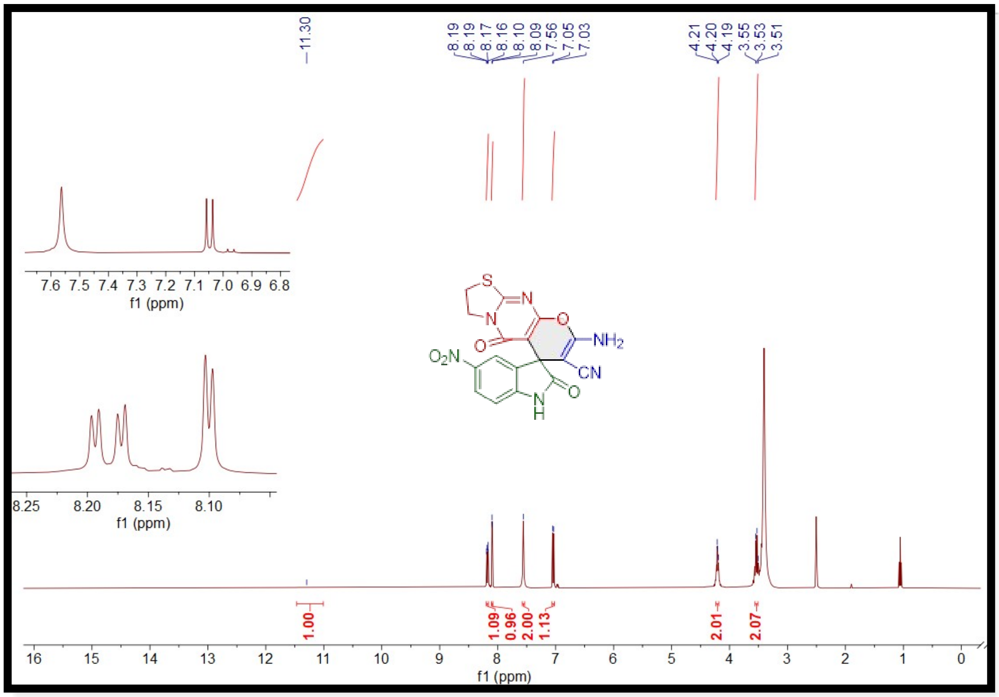

^{1}H-NMR of **4n**

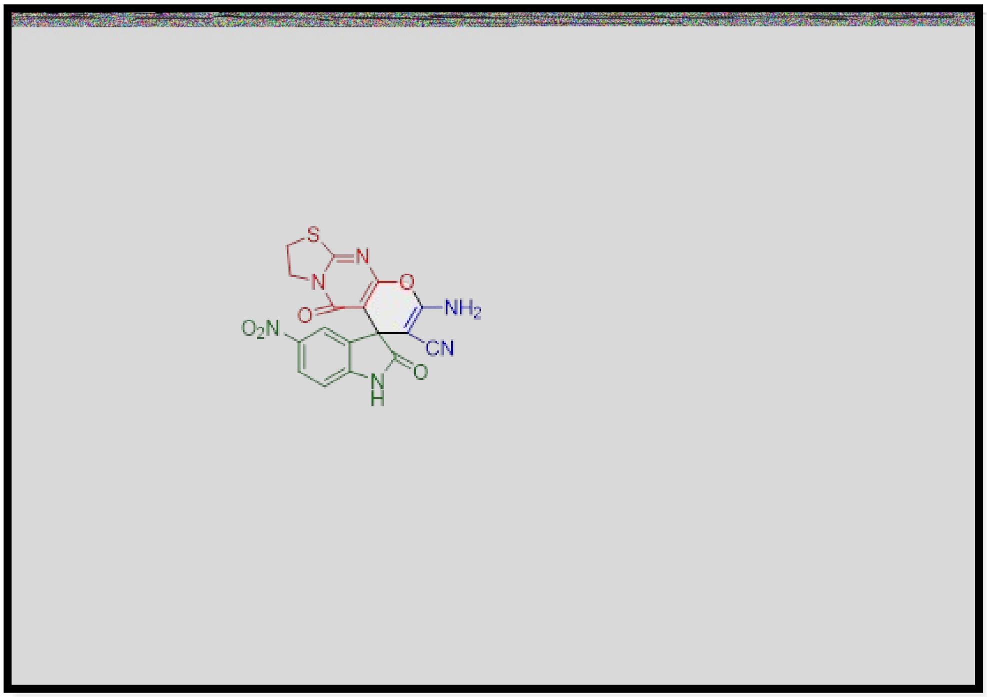

^{13}C-NMR of **4n**

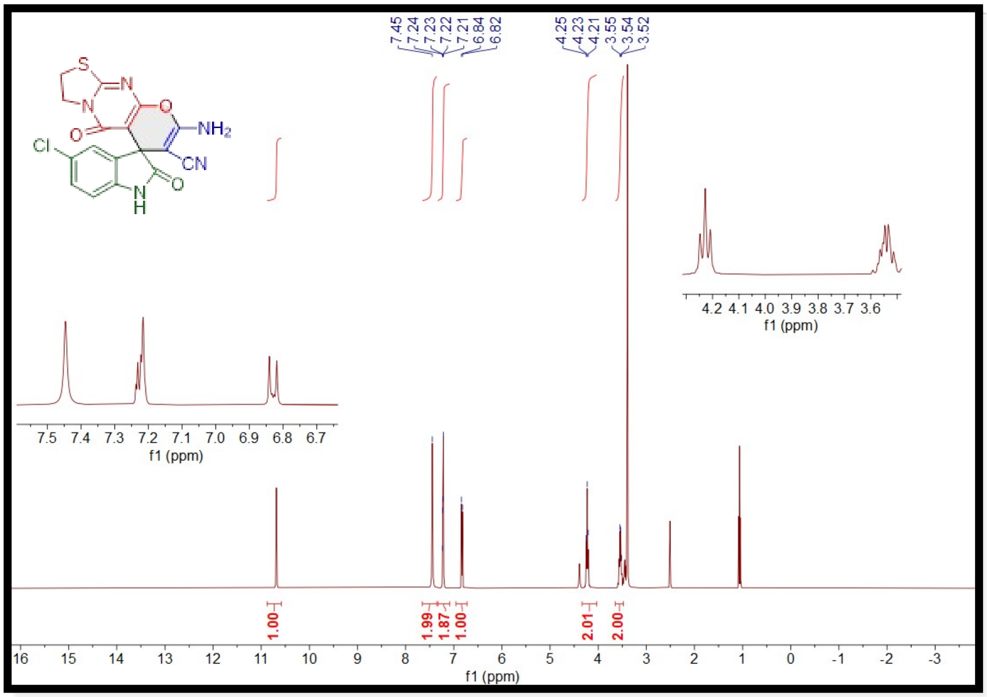

^{1}H-NMR of **4o**

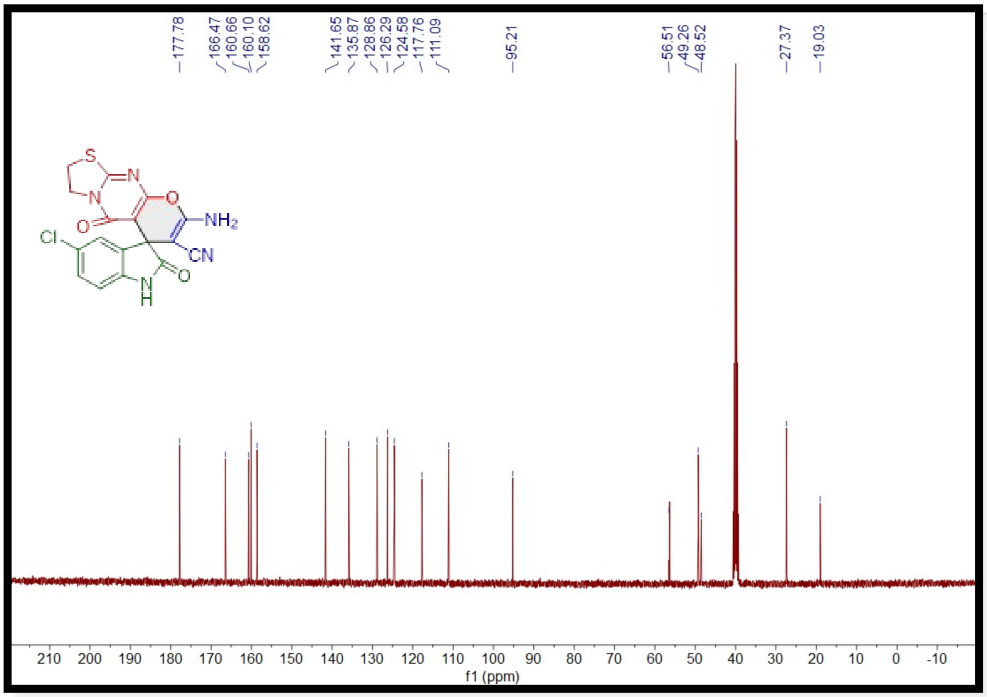

^{13}C-NMR of **4o**

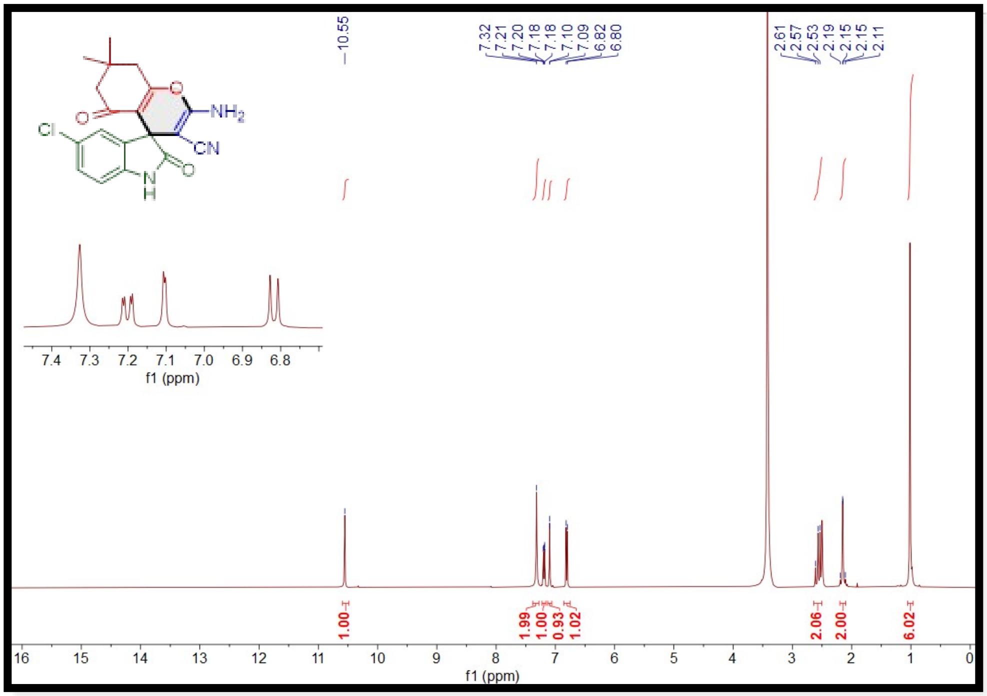

^{1}H-NMR of **4p**

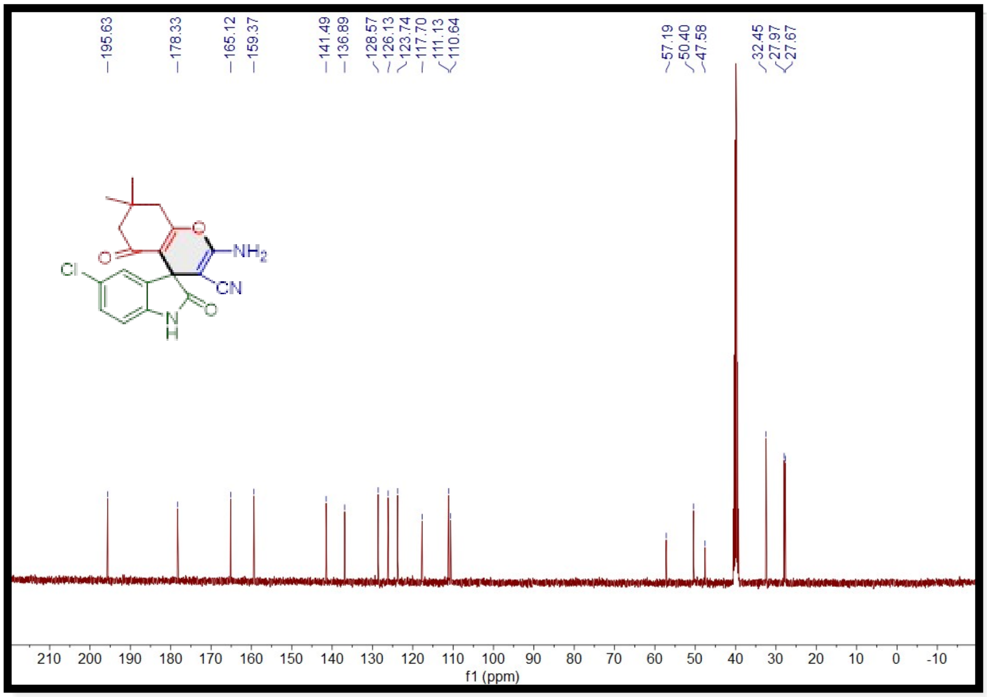

^{13}C-NMR of **4p**

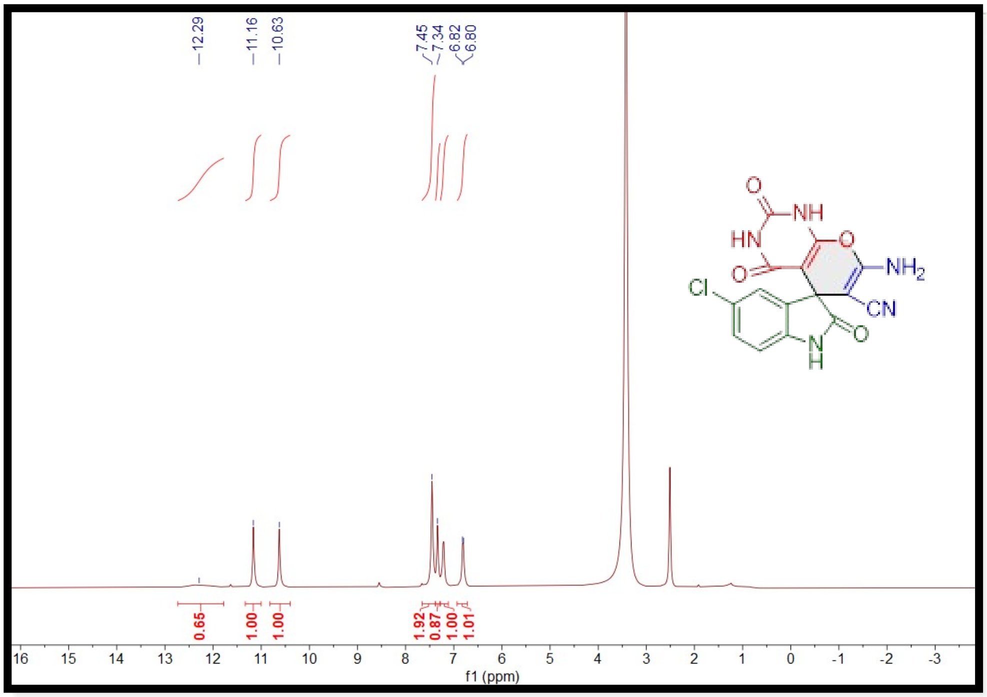

H-NMR of **4q**

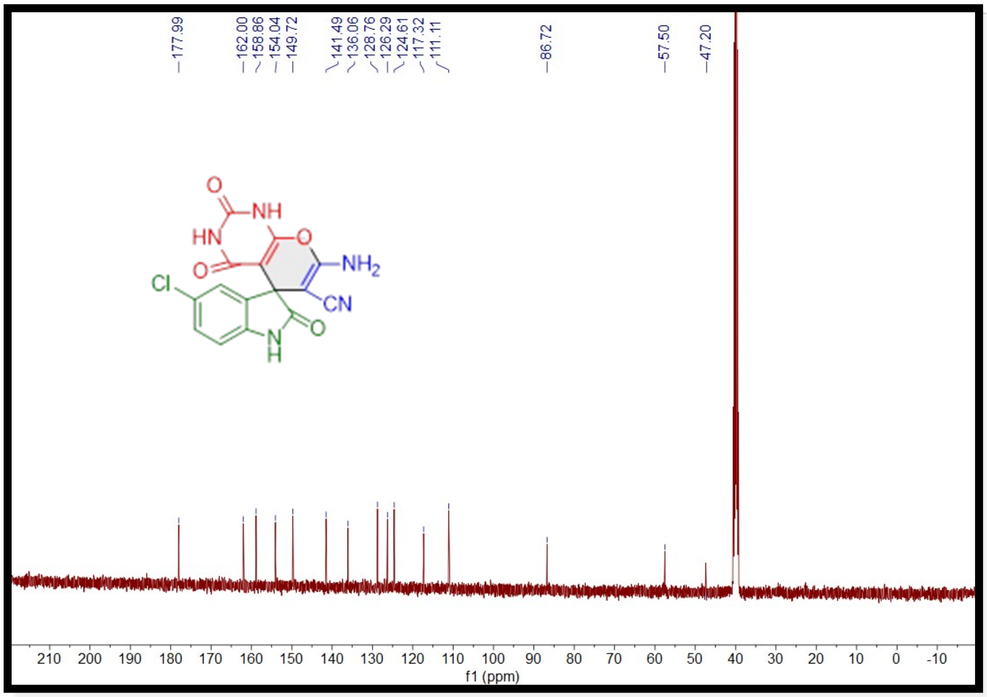

^{13}C-NMR of **4q**

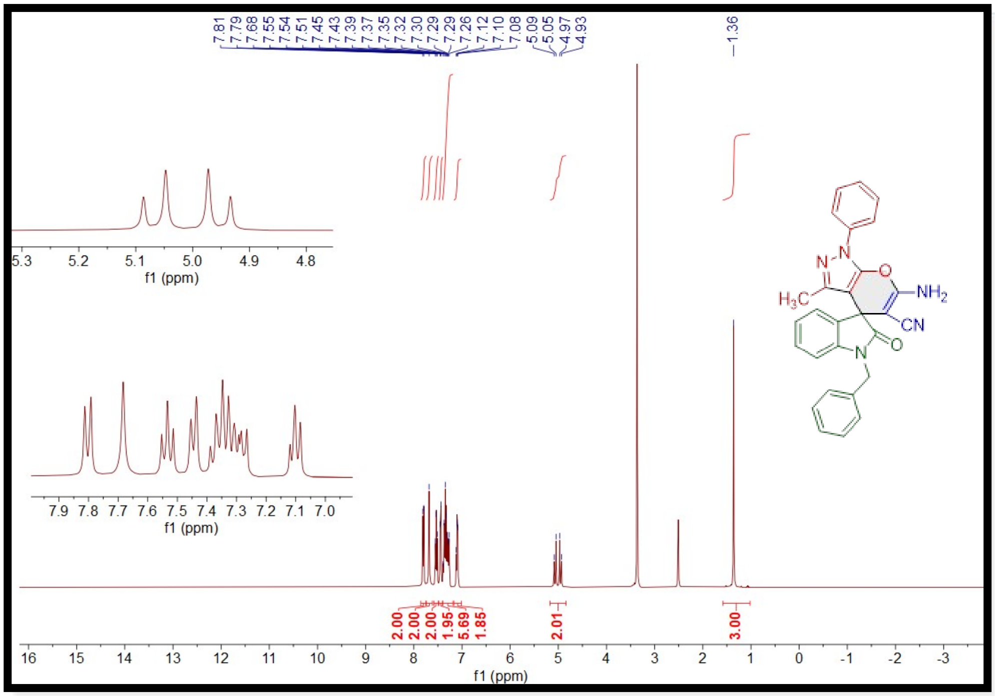

^{1}H-NMR of **4r**

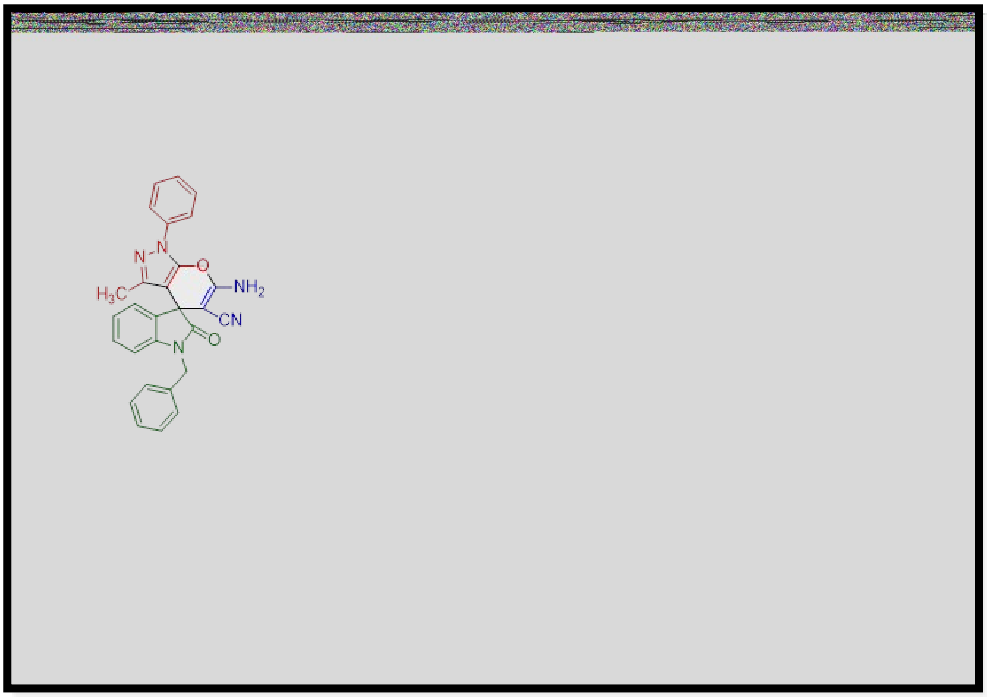

^{13}C-NMR of **4r**

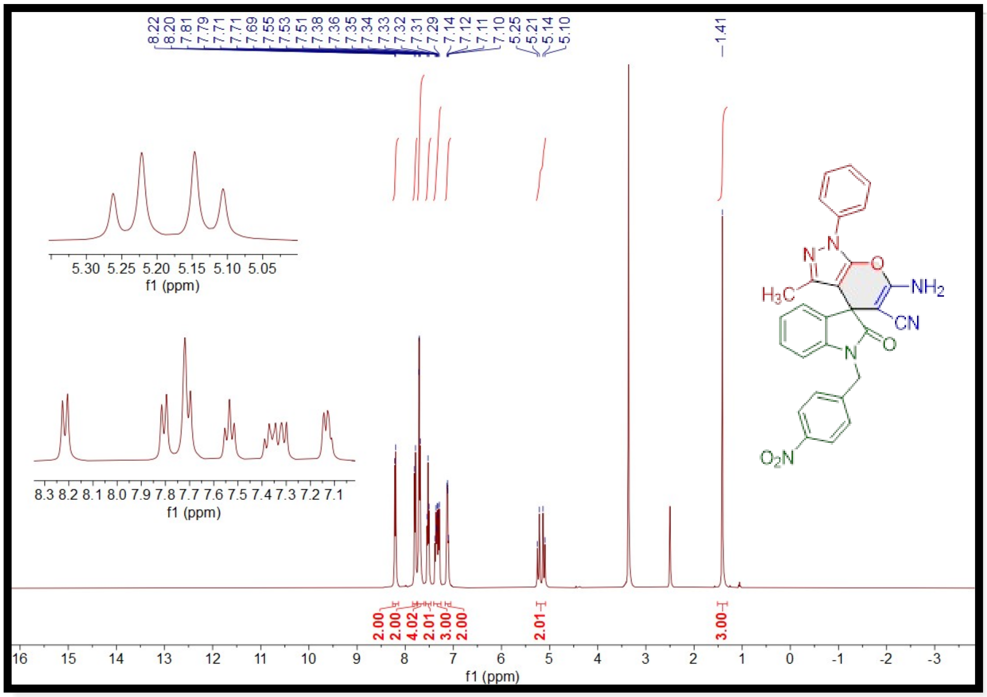

^{1}H-NMR of **4s**

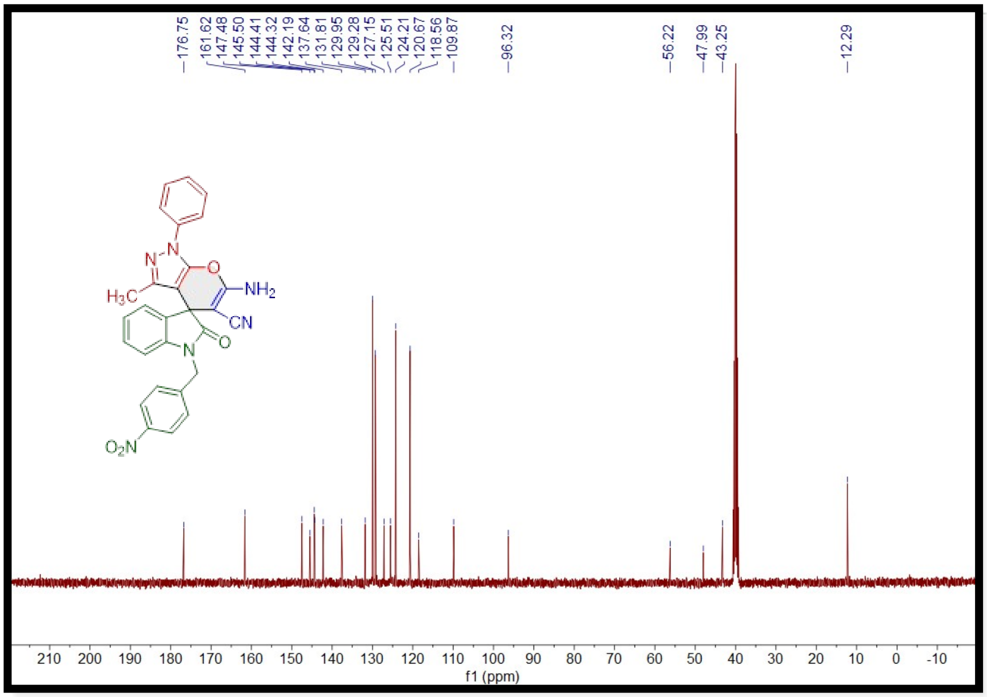

^{13}C-NMR of **4s**

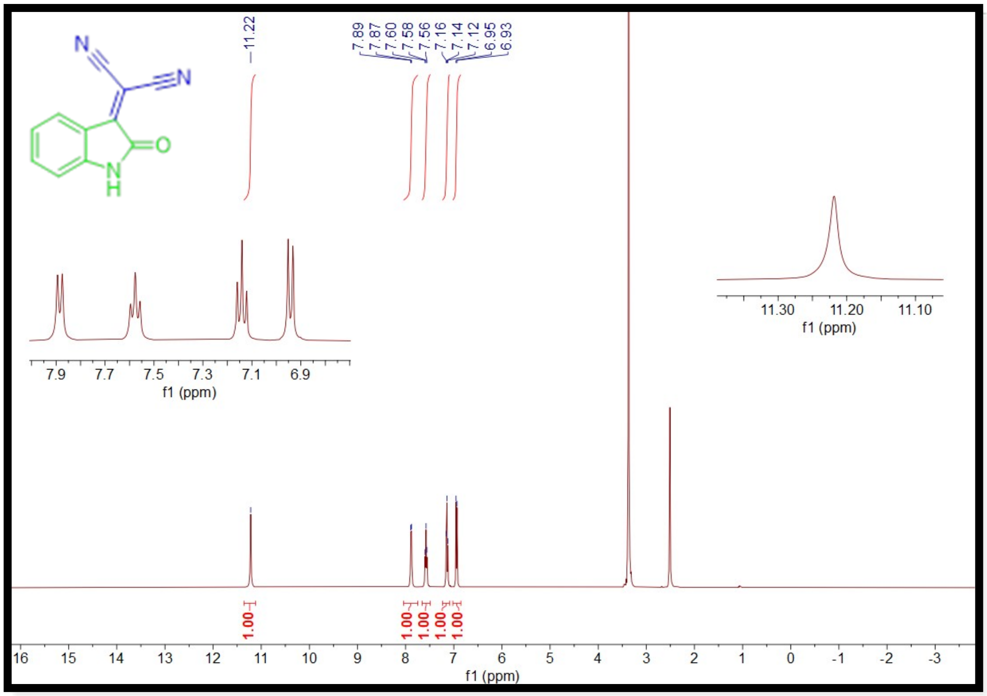

^{1}H-NMR of (**II**)

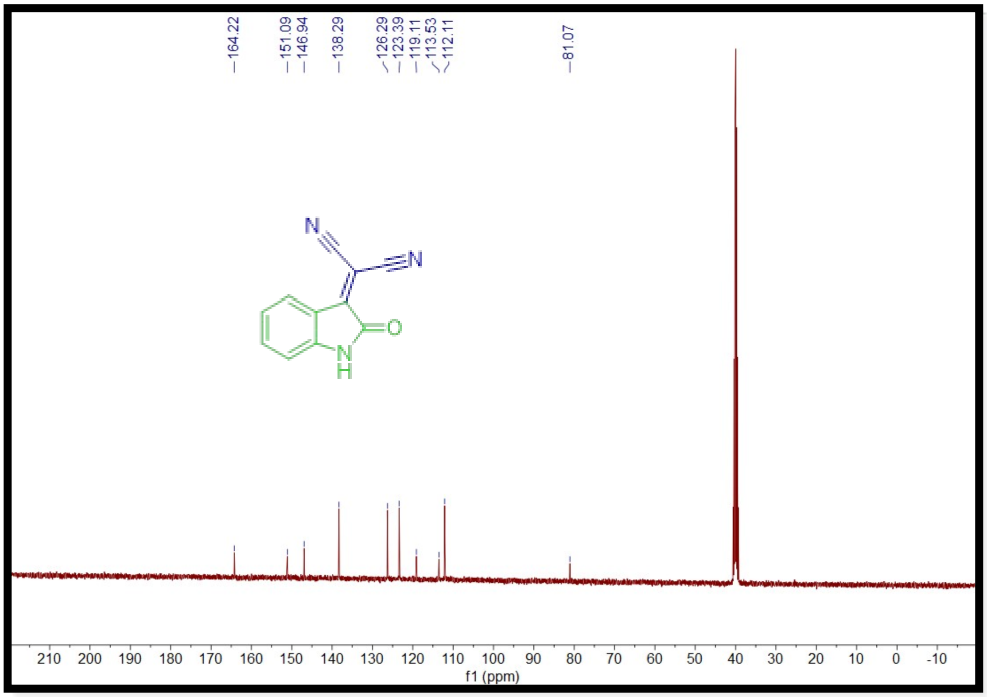

^{13}C-NMR of (**II**)
